# Role of π‑Spacers and Acceptors in Regulating the Photophysical Properties of BTPA Donor-Based Dyes: First-Principles Approach

**DOI:** 10.1021/acs.jpca.5c05194

**Published:** 2025-11-20

**Authors:** Juganta K. Roy, Asmita Adhikari, Tyler Lafferty

**Affiliations:** Clean Energy Materials Modeling Laboratory, Department of Chemistry and Physics, 14741 West Texas A&M University, Canyon, Texas 79016, United States

## Abstract

Dye-sensitized solar cells (DSCs) have the potential to access sunlight in the near-infrared (NIR) region. A precise understanding of the design and stability of photosensitizers is one of the ways to improve efficiency. Through this computational study, we systematically investigated the effect of π-spacers and acceptor groups on the photophysics of the BTPA-based dye to access photons from the NIR region. We incorporated two distinct acceptor groups, benzoic acid (BA) and cyanoacrylic acid (CA), with 20 different π-spacers such as BBT and iso-BBT fragments. Our calculation found that the asymmetry of the π-spacers is the decisive factor in lowering the energy gap. Thus, the bathochromic shift of the absorption peak is to the NIR region. Our result revealed that the maximum absorption peak was at 969 nm and broadened to 2000 nm. The perfect complementary absorption spectra and light-harvesting efficiency (LHE) curves of the designed dyes and XY1b dyes indicate their feasibility for the application in cosensitized DSCs. In addition, asymmetric π-spacers show a relatively lower intersystem crossing (ISC) rate, which increases the probability of charge transfer (CT) to the semiconductor and a higher excited-state lifetime. Designed dyes with BBT fragments show better intermolecular CT (ICT) properties, although the acceptor group does not affect the ICT. The results proved that the rational molecular design provides a valuable reference for synthesizing dyes with higher efficiency and can be explored further.

## Graphical abstract

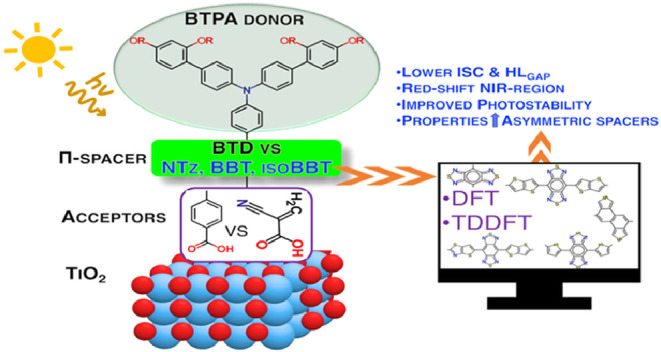

## 1 Introduction

With the growing population and technological advancements, global energy demand is projected to be 28 TW by the year 2050 from 13 TW in 2020.^1,2^ Governments, businesses, and individuals worldwide are increasingly seeking the economic, social, and environmental benefits of clean, zero-carbon electricity.^3^ Using renewable energy, such as solar energy,^4,5^ remains a crucial scientific and socioeconomic challenge. Photovoltaics such as dye-sensitized solar cells (DSCs) are pillars of the ongoing energy efforts to transition toward a zero-carbon emission society. DSCs venture to cheaply convert solar light in low-photon flux environments at a low light intensity and have shown promising photovoltages in the short-wavelength regions.^6,7^ Currently, DSCs reached a maximum efficiency of 15.2% with the donor *N*-(2′,4′-bis­(dodecyloxy)-[1,1′-biphenyl]-4-yl)-2′,4′-bis­(dodecyloxy)-*N*-phenyl-[1,1′-biphenyl]-4-amine (BTPA), leaving ample room for improvement in the sensitizer design.^8^

Dyes or photosensitizers with broader absorption that retain high quantum efficiency are urgently needed to progress the DSC field further.^4,9,10^ Significant efforts have been made to design novel photosensitizers due to the tunable nature of the photosensitizers through absorbing light in a particular region of the solar spectrum.^8,11–13^ The metal-free organic dyes could be considered as the donor (D)–acceptor (A)-based systems involving π-spacer units in between. Most photosensitizers have a large optical gap, causing low-energy photon waste and limiting the performance in full sun and low-light situations. Sensitizers that can harvest long-wavelength photons (>900 nm) and convert these photons into electricity are crucial to improving the DSC performance.^11^

Discovering building blocks with a red-shift behavior without harming the electron-transfer processes within the DSC device is crucial to modeling high-performance, long-wavelength-absorbing sensitizers. Triphenylamine (TPA) and its derivatives, such as BTPA and triazatruxene,^14^ are currently the leading donors with leading PCEs of 15.2 and 13.6%, respectively. BTPA over TPA efficiently reduces the charge recombination between the TiO_2_ surface and the redox shuttle, which helps to yield high *V*_OC_ values.^15^ Half of the total energy of sunlight is available in the NIR region^16^ and tailoring the sensitizers focusing in the NIR region is imperative for a higher PCE.^14,17^ Different approaches such as substituting π-spacers and the anchoring group of the BTPA-based dye have been reported.^10,12^ The cosensitization of the BTPA-based dye (MS5) with the spectral complementary dye has also been used to access the higher-wavelength photons.^8,13^ The cosensitization of MS5 (λ_max_ = 466 nm) with the wider-spectral-response dye XY1b (λ_max_ = 531 nm) produces a highly efficient and stable DSC with the PCE of 13.5% under standard AM1.5 G.^13^ MS5 dyes are modified to ZS10, ZS11, and ZS12 to obtain a red-shifted behavior, substituting the anchoring group benzoic acid (BA) with cyanoacrylic acid (CA) or 2-cyanobenzoic acid. Shen et al. reported CA-based MS5 dyes conducive to obtain red-shifted absorption spectra.^10^

Though π-spacers favor red-shifted absorption spectra due to their planarity,^18^ dyes with longer spacers, however, suffer from dye aggregation and charge recombination, limiting their PCE.^19^ Ren et al. reported that inserting comparatively shorter π-spacers in MS5 dyes improves the PCE.^8^ So, it is obvious to choose an optimal π-spacer with efficient ICT. In literature, many efficient π-spacers like naphthobisthiadiazole (NTz),^20^ benzo [1,2-c;4,5-c′]­bis­[1,2,5]­thiadiazole (BBT),^21^ and iso-BBT^21^ have been reported for their unique properties. The NTz unit shifts the absorption and emission spectra into the NIR region due to its shorter band gap. Compared to BBT, iso-BBT impacts the frontier molecular orbital energies by decreasing those of the HOMO and increasing those of the LUMO. Thus, the strong electron-accepting capacity of iso-BBT should enhance the *V*_OC_.^21^ Besides, the binding strength of the dyes to TiO_2_ is crucial for efficient electron injection and reducing the dye aggregation.^22^ The binding strength of the dye depends on the acceptor group; different acceptor groups have been reported with their merits. However, BA and CA groups are commonly used as dye anchors in DSCs for the TiO_2_ semiconductor.^23^

In this context, the present theoretical investigation aims to understand the BTPA-based dyes’ photophysics and design improved sensitizers using a state-of-the-art first-principles-based approach within the density functional theory (DFT) and time-dependent DFT (TDDFT). We have designed 40 D−π–A architecture-based dyes (Figure 1) by modifying the structure of the conventional dye, MS5, with a bulky BTPA donor, π-spacer 1,2,5-benzothiadiazole (BTD), and BA moieties and compared the geometric, optical, and charge-transport properties with MS5.^13^ The limited conjugation length and withdrawing strength of the BTD building block are design elements that can be significantly improved upon. Recently, the electron-withdrawing units of NTz,^20^ BBT, and iso-BBT^21^ have been examined as auxiliary acceptor-type building blocks for sensitizers in DSCs. Based on their performance, the BTD π-spacer in the MS5 parent dye is replaced with NTz, BBT, iso-BBT, and their derivatives to tune the photophysical properties and their feasible uses in DSCs. Also, substituting an S atom with a Se atom in the π-spacer can improve the PCE with its narrower band gap.^24^ The BBT and iso-BBT fragments extended using thiophene and theienothiophene are used to see the effect of lengthening conjugation.^25^

**Figure 1:**
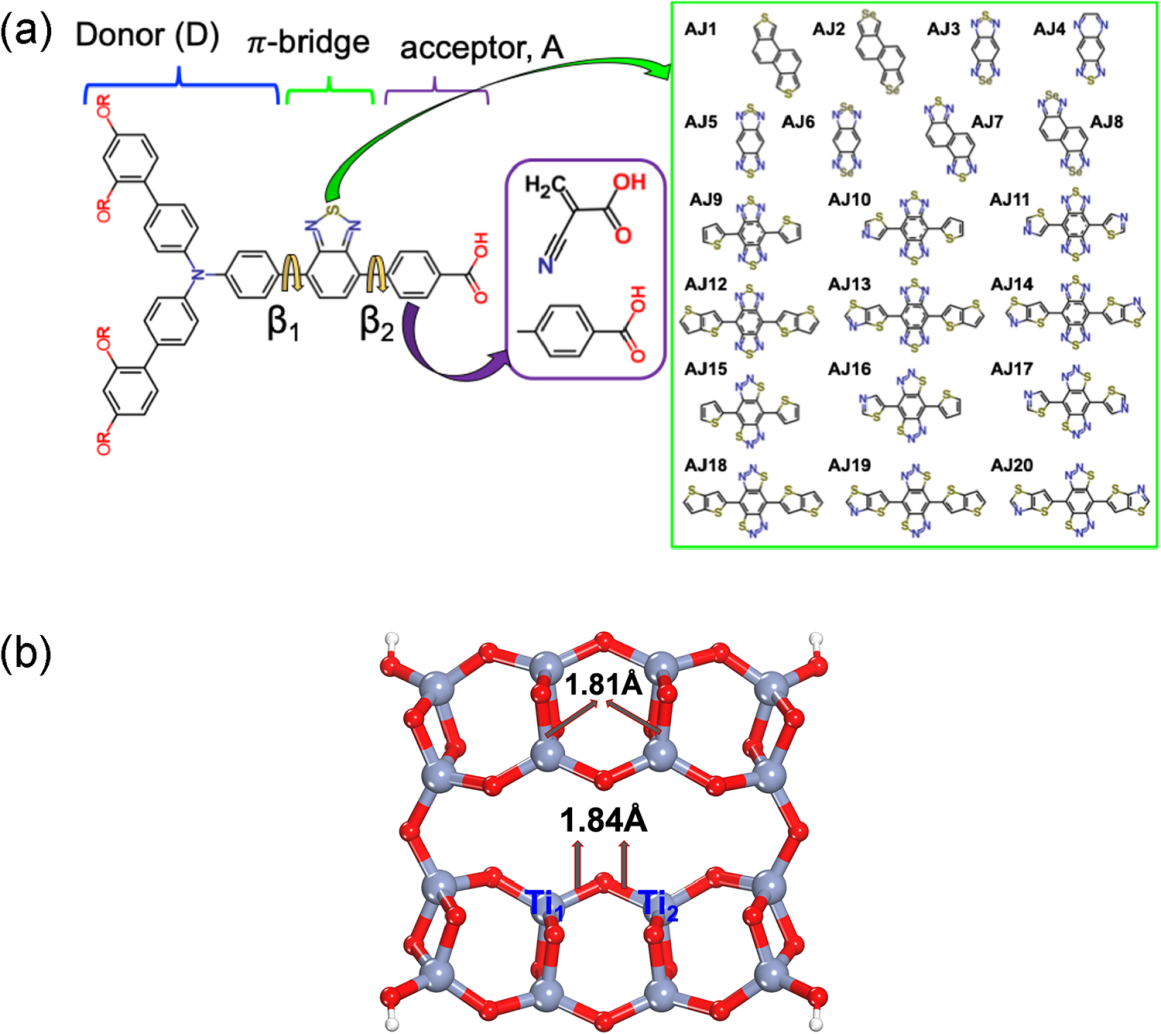
(a) Schematic of the design of the AJ dyes with the dihedral angles and library of π-spacers (green box). The representative sensitizer is the MS5 dye with the BTPA donor, BTD as the π-spacer, and benzoic acid as an acceptor. (b) Top view of the optimized geometry of the model anatase [(TiO_2_)_16_(H_4_O_2_)] cluster with important bond lengths (in Å) and two Ti atoms involved in bridged bidentate mode. Color code: bluish gray (titanium), red (oxygen), and white (hydrogen).

## 2 Computational Details

The calculations of designed AJ dyes and dyes bound to TiO_2_ (dyes@TiO_2_) were performed using DFT and TDDFT frameworks as implemented in the Gaussian16 package.^26^ The computational workflow used in this study to estimate different photophysical properties of the designed dyes is presented in Scheme 1. In all DFT and TDDFT calculations, we have incorporated the solvent effects using a conductor-like polarizable continuum model (CPCM)^27^ with tetrahydrofuran (THF) with a ε = 7.4257 solvent, similar to the experimental conditions for the MS5 dye. The ground-state geometry of the isolated dyes and dyes@TiO_2_ cluster was obtained using hybrid PBE0 functionals with the 6-31G­(d,p) basis set for C, N, O, and S atoms, while the effective core potential LANL2DZ and its corresponding basis set were used for Se and Ti atoms. Frequency calculations confirmed that the obtained structures have no imaginary frequency. The benchmarking calculation for the ground-state functional of MS5 dyes is described in Figure S1. We found that a hybrid functional like PBE0 for the ground state, as it is better to reproduce the experimental geometrical features,^28^ is closely matched with the experimental energy gap of FMOs and their positions in the energy-level diagram.

**Scheme 1:**
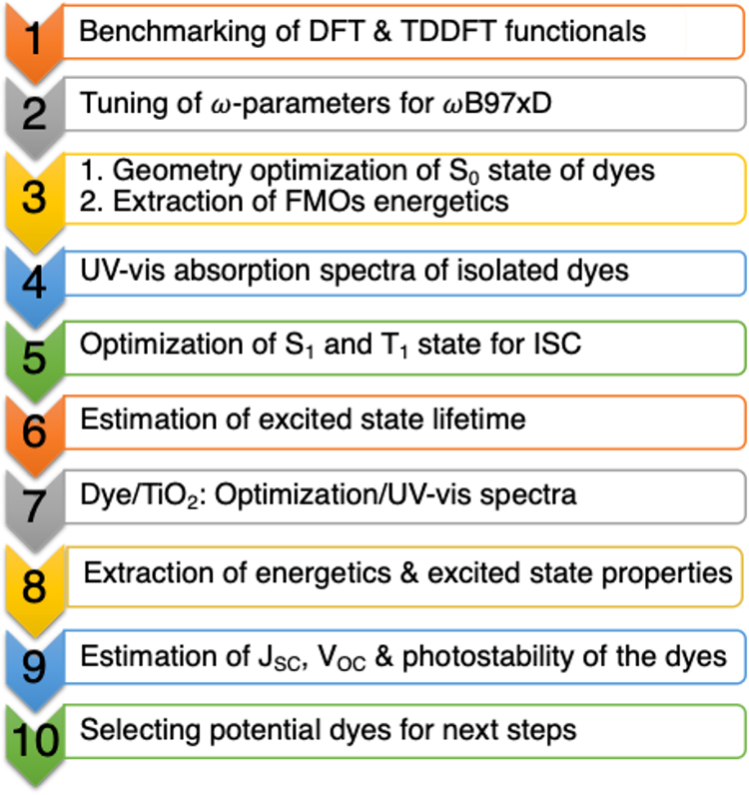
Computational Workflow for Estimated Photophysical Properties and Device Parameters of the Designed AJ Dyes

After the ground-state geometry was found, TDDFT single-point calculations were performed with the solvent-phase DFT-optimized geometry. To obtain vertical excitation energies and oscillator strengths (OSs) of the dyes and dyes@TiO_2_ cluster, the lowest 50 singlet–singlet transitions of the optimized ground-state geometry in the solvent phase were selected. We used different hybrid functionals like B3LYP, CAM-B3LYP,^29^ PBE0,^30^ M062X, LC-ωPBE, BhandH, TPSSh, and ωB97XD^31^ functionals using the same basis set to confirm the experimental absorption profile, specifically the maximum absorption peak (λ_max_) of the MS5 dye. The results are outlined in Figure S2a, and we found that the ωB97XD hybrid functional will be suitable to simulate the absorption of MS5, as it has been found that the ωB97XD functional reproduces the experimental observations like the absorption phenomenon for the conjugated system.^32,33^ ωB97XD is a range-separated version of Becke’s 97 functional with additional dispersion correction and its default range separation parameter value of ω = 0.2.^31^ Range-separated hybrid functionals are important for orbital energy modeling of conjugated molecules that involve charge transfer excitation, and it can be optimized by tuning ω, adjusting the degree of long-range interactions included in our calculations.^34^ We tuned ω from 0.01 to 0.25 with an increment of 0.05 and found that 0.1 Bohr can perfectly reproduce the λ_max_ of the MS5 dye (Figure S2b). An optimally tuned (OT)^33,35,36^ ω value has been used for all of the TDDFT calculations of designed AJ dyes and dyes@TiO_2_. The TDDFT method can be written as DFT/ωB97XD/6-31G­(d,p)//OT-ωB97XD/6-31G­(d,p). The first excited-state geometry and the triplet-state geometry of the AJ dyes are optimized using the OT-ωB97XD/6-31G­(d,p) level of theory, respectively, to compute the nonradiative intersystem crossing (ISC) rate. A description of the ISC calculation can be found in Section 3.6.

For a deeper understanding of the interfacial photophysical properties of the adsorbed dyes on a semiconductor, the anatase TiO_2_(101) surface model cluster [(TiO_2_)_16_(H_4_O_2_)] was constructed by cutting from an anatase (101) surface. The latter was built replicating (5, 3, 2) the unit cell by means of the VESTA program, as described elsewhere.^37,38^ This cluster model has been successfully studied for the adsorption of different dyes.^37–40^ Although the cluster model is not perfect for global description and suffers from finite-size effects, we implemented this model to screen photoefficient AJ dyes by scrutinizing the electrochemical parameter in a fast and efficient way. However, finite-size effects in a small cluster can be improved by adopting a self-assembled structure of the small clusters.^41^ However, Persson et al.^42^ explained that a stoichiometric small cluster like (TiO_2_)_16_ with proper charge distribution gives reasonable results without showing significant discrepancies compared to large models.^43,44^ A small cluster like (TiO_2_)_16_ usually gives rational outcomes in small-scale theoretical models and exhibits insignificant variances with large and periodic models.^42,45,46^ In all of our cluster calculations, the atoms in the cluster are fully relaxed to accurately describe the electronic structure after the adsorption of dyes.

## 3 Results and Discussion

### 3.1 Ground-State Structure of Dyes

Ground-state geometries of the designed dyes were obtained using the PBE0/6-31G­(d,p) level of theory. 40 dyes were designed based on the MS5 dye,^13^ which consists of a triphenylamine-based donor BTPA, a BTD π-bridge, and the benzoic acid (BA) acceptor group. Designed dyes consist of different π-bridges and two different acceptor groups. The list of different π-bridges is illustrated in Figure 1, while the optimized ground-state geometries of the dyes are presented in Figure S3. Those π-bridges have been used in the literature due to their enhancing optoelectronic properties.^47–49^ Also, acceptor groups, like cyanoacrylic acid (CA) and BA, have been used to increase the photoconversion efficiency of solar cells.^10^ Structural attributes like dihedral angles between (1) the donor and π-bridges and (2) the π-bridges and the acceptor group play a key role in achieving a smooth CT from D to A to a semiconductor like TiO_2_. Even though π-bridges are approximately planar, the dihedral angles (β_1_) between the D- and π-bridges need to be assessed, as the ICT depends on the planarity of dyes. The bond length between D and π-bridges is almost the same for all of the designed dyes (1.47 Å), indicating similar strength of interaction. The π-bridge is connected directly to the acceptor group (BA or CA). The dihedral angle (β_2_) and bond length between the fragments assessed the effectiveness of the ICT between the donor and the semiconductor. All of the dihedral angles for the designed AJ dyes are summarized in Table S1.

Dihedral angles, β_1_ and β_2_, are almost similar for dyes from AJ1 to AJ8, and the average value is 140°. However, the dyes from AJ9 to AJ20 vary, depending on the position of the dihedral angle and the type of acceptor groups. Although β_1_ is almost similar for both BA and CA acceptor groups, β_2_ is more planar in the case of CA compared to BA. Besides, the symmetrical distribution of nitrogen atoms in the 5-membered rings makes the β_2_ planar (AJ11, AJ14, AJ17, and AJ20) for the designed dyes. Overall, the dyes with CA exhibit greater planarity compared to those with BA, and this could be due to the spatial freedom of a smaller acceptor region. This planarity enhances the ICT from the conjugation bridge to the acceptor groups and the semiconductor. The increased ICT of CA compared to BA can be reasoned through inductive effects, stemming from the C≡N bond, and can facilitate electron movement from the donor into the acceptor moieties. All of the designed dyes showed planar or near-planar geometry starting from D to π-bridge to A. Moreover, our calculation found that replacing a 6-membered ring with a 5-membered ring favors the ICT as the dihedral angle is almost planar.

### 3.2 Energetics and Electron Distribution Map of the Dyes

The efficiency of the sensitizers, which largely depends on the frontier molecular orbital (FMO) energetics, is critical to accessing the higher-wavelength photons from the solar spectrum. Energy-level alignment of FMOs plays a key role in harvesting photons and dye regeneration. According to the DSC’s working principle, the higher occupied molecular orbital (HOMO) and lowest unoccupied molecular orbital (LUMO) energy levels of dyes should be lower than the redox potential of the iodine electrolytes (−4.8 eV) to ensure the dye regeneration and higher than the conduction band (CB) minima of TiO_2_ (−4.0 eV) to maintain the efficient electron injection, respectively. Computed HOMO and LUMO energies are outlined in Figure S4. Although the energies of the HOMO are almost the same, the LUMO energies change based on the type and length of conjugation. The LUMO energies of CA are lower than the homologous BA-containing dyes. It might be due to the (1) strong electron-withdrawing characteristics of the CA group, stabilizing the LUMO by lowering the energy, and (2) the bulkiness of BA, which makes it less efficient than the sleek CA acceptor. All of the designed dyes exhibit lower LUMO energies except AJ1 and AJ2 compared to MS5.

The energy gap (E_gap_) between the HOMO and LUMO energies of the designed dyes is computed and is outlined in Figure 2. The dyes with CA acceptors have a lower E_gap_ than those of the dyes with BA acceptors. AJ6, AJ9, AJ10, AJ11, AJ12, AJ13, and AJ14 dyes have lower E_gap_ values and all of the dyes contain BBT as the core π-spacer. Beyond the donor of the sensitizers, E_gap_ depends on the conjugation length and the substituents on the conjugation, which are responsible for the red-shifted behavior of the absorption spectra. The π-conjugation length was altered through the different fused 5- or 6-membered rings. Nitrogen electron-donating groups were substituted into the π-system to increase the electron density and lower the E_gap_. Nitrogen has a lone-pair electron that can undergo an *n* → π transition. Heteroatoms in the conjugation affect the E_gap_ such as AJ1 vs AJ7 and AJ2 vs AJ8. In both instances, the N atom helps to reduce the E_gap_ compared to the S/Se atom by lowering their LUMO. Thiophene is a commonly used substituent that increases the power conversion efficiency by well over 7% for photovoltaic devices. It has suitable HOMO energy levels, and thus, E_gap_ enables a more π-extended conjugated backbone.^50^ However, selenophenes improve charge mobility^50,51^ and enhance the photovoltaic devices’ efficiency without raising carbon content.^52^ Structures with more Se (AJ1 vs AJ2, AJ5 vs AJ3 and AJ6, and AJ7 vs AJ8) have lower energy gap values than others due to the lowering of the LUMO levels.

**Figure 2:**
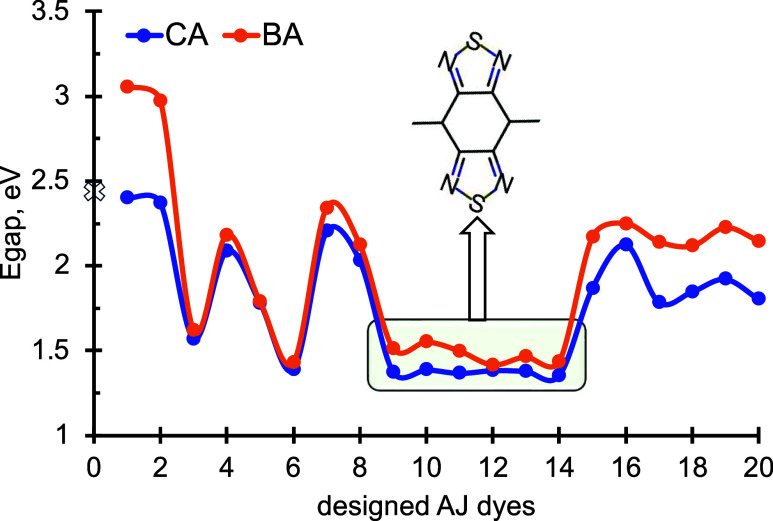
HOMO–LUMO gap (E_gap_) of the designed AJ dyes with the PBE0/6-311G­(d,p) level of theory. The lowest gap is obtained from AJ9 to AJ14, the dyes containing the BBT core as their conjugation.

Our results showed that the planarity of the molecule is the most determinative factor compared to the π-conjugation length for lower *E*_gap_ values. Also, the N heteroatom contributes to a limited extent in lowering the *E*_gap_ by donating electrons to the π-system. The intrinsic electronic structures of the dyes such as the FMO electronic density distribution map and their energetics potentially affect the electronic transfer and excitation characteristics of the dyes. Figure 3 (Figures S5 and S6) depicts the density maps of important FMOs, including the HOMO, LUMO, and LUMO+1, of the isolated dyes in THF. FMO can be used to evaluate the ICT phenomenon and the CT state. The HOMO of all of the dyes is distributed over the donor BTPA and π-fragments to some extent up to the acceptor group. At the same time, the LUMO is centered either on the π-fragments (BA) and then transferred to the acceptor group in LUMO+1 or on π-fragments (CA) and some extent to the acceptor group and in LUMO+1 centered on the acceptor group. The distribution exhibits an evident phenomenon of electron density separation, which is conducive to moving photogenerated electrons from D to A within the molecule. These molecules have optimized charge transfer properties by incorporating the CA acceptor group and the most optimized geometry for extracting using the MS5 derivative for the π-conjugation scheme. Exciton binding energy (*E*_b_), the energy required to separate an exciton into its constituent electron and hole, also affects the ICT process directly and hence it is an important factor for the efficiency of DSCs.^53^ The value of *E*_b_ can be estimated by the energy difference between the electronic and optical band gaps (*E*_b_ = Δ*E*_HL_ – *E*_*S*1_),^38,54^ where the first term is *E*_gap_ and the second one is the first singlet excitation energy of the isolated dye. The negative value of *E*_b_ indicates that the exciton pairs recombine with each other, while positive values refer to the exciton pairs being separated; separation of exciton pairs is essential to increase the quantum yield of the device.^53^ The computed values of *E*_*b*_ are tabulated in Table 2. Most AJ dyes, specifically AJ3, AJ5, and AJ9-AJ14 for both acceptor groups (BA and CA), show sufficient energy to not recombine the exciton pairs and increase the efficiency of DSC devices.

**Figure 3:**
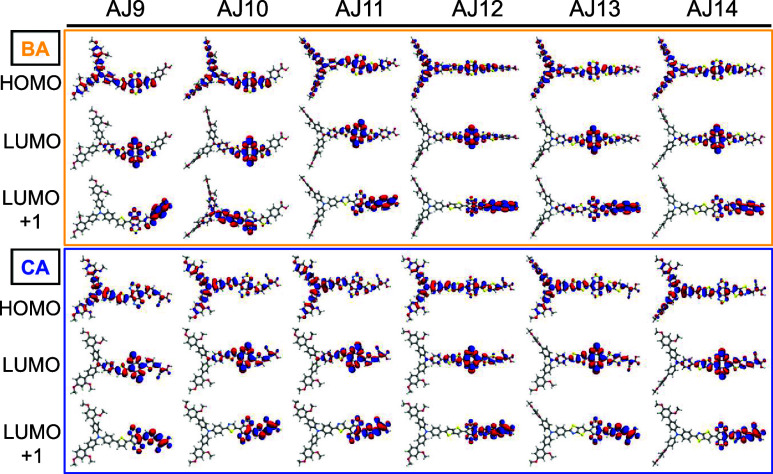
HOMO–LUMO electron density map of the designed dyes calculated in a THF solvent. Yellow and blue boxes indicate the benzoic acid and cyanoacrylic acid anchoring group, respectively. The first row of each box is the HOMO, and the second and third rows represent the LUMO and LUMO+1, respectively.

### 3.3 UV–Vis Absorption Spectra of Isolated Designed Dyes

To verify whether the designed dye molecules can achieve the expected degree of spectral match, the simulated UV–vis absorption spectra of all the dyes were calculated using THF solvent and are shown in Figure 4 (Figure S7). Also, the relevant spectral parameters are presented in Tables 1 and S2. One of the most important factors of the isolated dyes is to achieve a higher degree of spectral match to increase the PCE of the DSCs. UV–vis spectra of all of the AJ dyes show a red-shifted behavior compared to MS5, which suggests that the designed dyes show potential for the DSC device to access photons to NIR regions of the solar spectrum.

**Figure 4:**
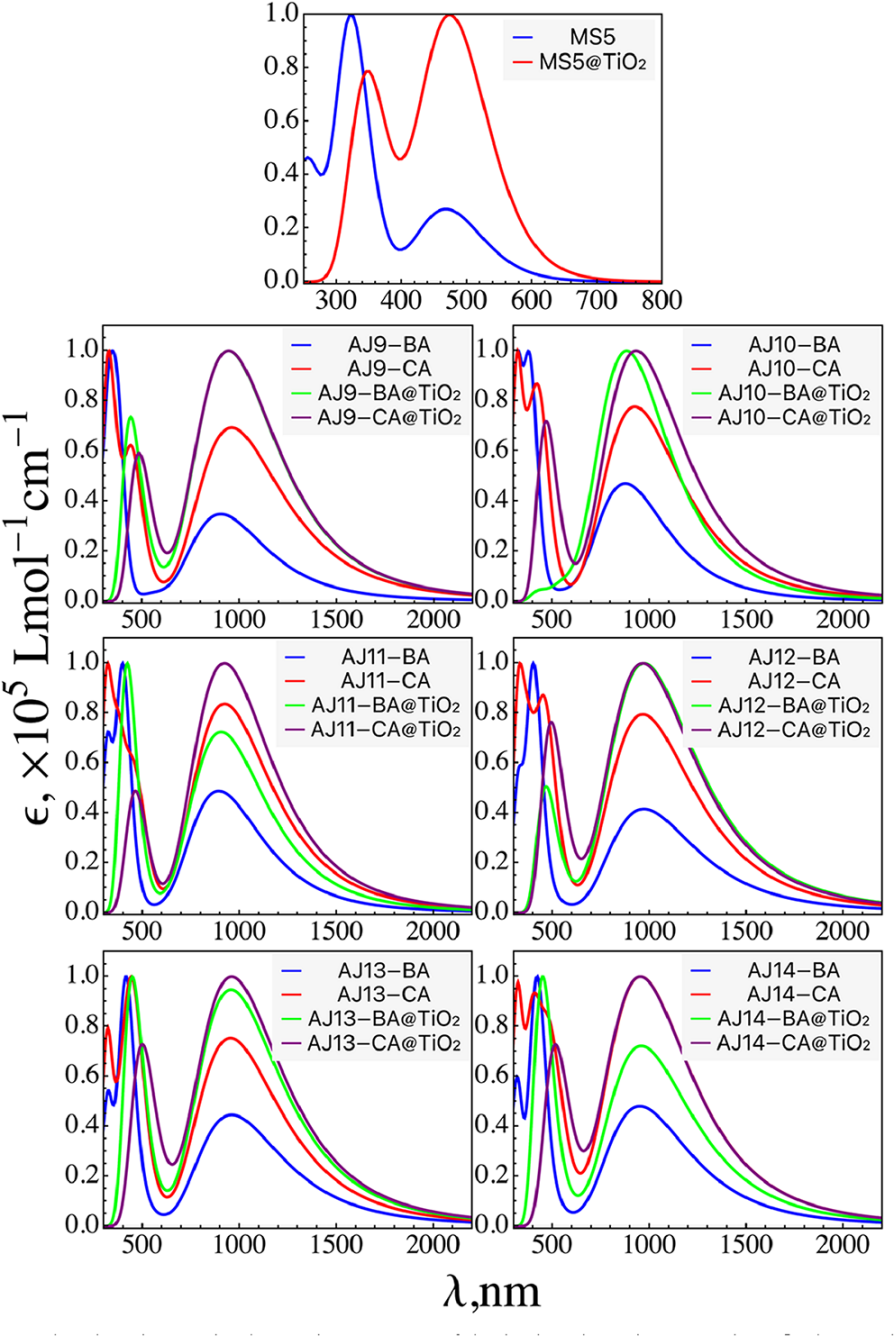
Simulated UV–vis absorption spectra of the isolated AJ dyes, AJ dyes@TiO_2_, and the MS5 reference dye in the THF solvent simulated at the TDDFT/CPCM/OT-ωB97XD/6-31G (d,p) level of theory. For Se and Ti heavy atoms, the LANL2DZ basis set was used.

**Table 1:** Excited-State Properties (Maximum Wavelength in nm, Oscillator Strength, Transition State, and Major Transition of the FMOs with Their Contribution) of the Isolated Designed AJ Dyes with Lower E_gap_ Values Computed at the TDDFT/CPCM/OT-ωB97XD/6-31G­(d,p) Level of Theory in the THF Solvent^a^

name	transition	major contribution (%)	λ_max_ (nm)	OS
AJ9-BA	S_0_ → S_1_	H → L (75.2%), H^–1^ → L (24.4%)	903.41	0.7071
	S_0_ → S_7_	H → L^+3^ (77.7%), H → L^+2^ (5.4%)	371.95	1.4325
AJ10-BA	S_0_ → S_1_	H → L (72.7%), H^–1^ → L (26.7%)	874.85	0.7389
	S_0_ → S_5_	H → L^+1^ (74.9%)	388.53	1.0046
	S_0_ → S_6_	H→ L^+3^ (42.5%), H^–1^ → L^+3^ (17.8%)	382.62	0.3766
AJ11-BA	S_0_ → S_1_	H → L (73.5%), H^–1^ → L (25.5%)	891.33	0.9156
	S_0_ → S_4_	H→ L^+1^ (35.0%), H → L^+2^ (24.1%), H^–1^ → L^+1^ (20.4%)	398.18	1.7026
AJ12-BA	S_0_ → S_1_	H→ L (78.7%), H^–1^ → L (20.1%)	969.46	1.0280
	S_0_ → S_6_	H→ L^+1^ (34.0%), H → L^+2^ (32.7%), H^–1^ → L^+1^ (12.8%)	404.07	1.8112
AJ13-BA	S_0_ → S_1_	H → L (81.2%), H^–1^ → L (17.4%)	957.85	1.0631
	S_0_ → S_6_	H → L^+2^ (39.9%), H → L^+1^ (38.9%)	412.54	2.1041
AJ14-BA	S_0_ → S_1_	H → L (73.4%), H^–1^ → L (25.2%)	948.91	1.1044
	S_0_ → S_5_	H^–8^ → L (39.5%)	421.18	0.8043
	S_0_ → S_6_	H^–8^ → L (25.5%), H → L^+2^ (21.7%), H → L^+1^ (21.3%)	418.63	1.2879
AJ9-CA	S_0_ → S_1_	H → L (77.5%), H^–1^ → L (21.3%)	958.81	1.1038
	S_0_ → S_3_	H → L^+1^ (49.8%), H^–1^ → L^+1^ (33.4%)	452.12	0.8130
AJ10-CA	S_0_ → S_1_	H → L (73.1%), H^–1^ → L (25.1%)	923.88	1.1014
	S_0_ → S_3_	H → L^+1^ (25.9%), H^–1^ → L^+1^ (21.6%), H^–6^ → L (15.8%)	441.95	0.4822
	S_0_ → S_4_	H^–3^ → L (22.1%), H → L^+1^ (21.6%), H^–6^ → L (19.0%)	437.81	0.4588
AJ11-CA	S_0_ → S_1_	H → L (72.7%), H^–1^ → L (24.9%)	921.40	1.1499
	S_0_ → S_3_	H → L^+1^ (47.7%), H^–1^ → L^+1^ (40.0%)	463.44	0.6852
	S_0_ → S_7_	H → L^+2^ (66.3%)	381.24	0.4022
AJ12-CA	S_0_ → S_1_	H → L (66.8%), H^–1^ → L (31.2%)	964.78	1.2900
	S_0_ → S_4_	H → L^+1^ (46.3%), H^–1^ → L^+1^ (40.5%)	463.15	1.2061
AJ13-CA	S_0_ → S_2_	H → L (70.0%), H^–1^ → L (27.5%)	952.41	1.3181
	S_0_ → S_20_	H → L^+1^ (27.5%), H^–1^ → L^+1^ (21.5%), H^–4^ → L (20.3%)	459.23	0.9403
	S_0_ → S_21_	H^–4^ → L (22.6%), H → L^+1^ (21.2%), H^–1^ → L^+1^ (15.4%)	457.56	0.4158
	S_0_ → S_31_	H → L^+2^ (41.7%)	397.61	0.4870
AJ14-CA	S_0_ → S_1_	H → L (68.2%), H^–1^ → L (29.1%)	952.92	1.3881
	S_0_ → S_3_	H → L^+1^ (49.6%), H^–1^ → L^+1^ (38.5%)	490.77	0.9398
	S_0_ → S_8_	H → L^+2^ (80.5%)	400.53	1.0143

a The transition was considered with OS ≥ 0.3 and λ_max_ ≥ 370.

The spectra of these dyes have two distinct absorption bands in the UV and visible regions. If we examine the absorption spectra of MS5 and all designed dyes, MS5 has a very low-intensity absorption peak between 400 and 600 nm, while almost all of the AJ dyes have a very high-intensity absorption peak in this region that is extended to the NIR region. It is noted that the spectral match of XY1b and AJ dyes is in the UV and NIR regions of the spectra. Combining the UV- and NIR-absorbing dyes with complementary absorption spectra, cosensitization strategies can be adopted to develop panchromatic DSCs that cover the full solar irradiance spectrum. In summary, XY1b and the designed AJ dyes exhibit perfect spectral complementarity. Theoretically, their combined absorbance range covers the entire visible region to the NIR region. Dyes with the CA acceptor group show a bathochromic shift compared to the BA acceptor group.

### 3.4 Adsorption of Dye Molecules on the (TiO_2_)_16_ Cluster

Experimental and computational studies have shown three different types of binding modes of a dye to the TiO_2_ surface such as monodentate, chelated, and bridged bidentate modes.^37,55–57^ Bridge bidentate mode has been demonstrated by Fourier transform infrared (FTIR) spectroscopy and surface-enhanced Raman spectroscopy (SERS) tests to allow the most stable and preferable binding mode of the carboxyl groups to the TiO_2_ surface.^58,59^ In literature, chelated binding mode is reported only for the catechol-like^60^ anchoring groups, which are different from our studied anchoring groups. As this work is not intended to screen the potential candidate based on comparative adsorption energy, we reported the most stable and preferable binding mode of the carboxyl group. The adsorption of the dye on the (TiO_2_)_16_ cluster is achieved in the form of the bidentate bridging manner, which is manifested by the formation of Ti–O covalent bonds between the two O atoms on the carboxyl group of the dye and the two Ti atoms on the (TiO_2_)_16_ cluster, respectively, while the protonated H atom in the carboxyl group is transferred to the double-coordinated surface O atom nearby. The optimized structures of designed AJ dye–(TiO_2_)_16_ complexes are shown in Figure S8. The calculated average Ti–O distances in all of the dye–TiO_2_ complexes are within 2.00 to 2.04 Å, comparable to the Ti–O bond length in bulk TiO_2_ (1.934 ∼ 1.980 Å), confirming that chemisorption of the dyes on the TiO_2_ (101) surface occurs, which facilitates the charge injection of photogenerated electrons to the semiconductor TiO_2_. Moreover, to explore the stability of the structure of designed dye–(TiO_2_)_16_ models, the adsorption energy (*E*_ads_) of the dye adsorbed on the (TiO_2_)_16_ cluster was calculated by the following equation:

$$E_{\mathrm{ads}}=E_{\mathrm{dye@Ti}O_{2}}-E_{\mathrm{dye}}-E_{\mathrm{Ti}O_{2}}$$

The DFT-computed adsorption energy lies between −1.99 and −0.35 eV in the case of BA, whereas it lies between −2.14 and −0.13 eV for the CA acceptor group. All of the adsorption energies are tabulated in Table 2. AJ2, AJ3, AJ6, and AJ8 show the highest adsorption energies. Surprisingly, all four dyes with the highest value of *E*_ads_ contain a Se atom in their π-spacer. The Se atom has greater polarizability and its outermost electron is larger and more loosely distributed than those of other heteroatoms.^61^ Those unique properties affect the electronic structure of the dye–TiO_2_ interface and help to achieve tighter binding.^62^ AJ14-CA dyes show the lowest adsorption energy, −0.13 eV. In most cases, the CA group shows higher adsorption energies compared to the BA group. A longer acceptor group has been found to allow strong adsorption and a higher PCE.^63^ Additionally, charge transfer will be more efficient if there is a relatively strong electronic coupling between the adsorbed dye and TiO_2_ due to interactions of the dye acceptor group, and TiO_2_, the substrate, is shown via the LUMO orbital position. Qualitative coupling could be estimated by reducing the LUMO energy level of the adsorbed dye.^64,65^ In this study, all bound dyes (except AJ12-BA and AJ9-CA) show decreased LUMO energy levels compared to their isolated LUMO energy, indicating the possibility of efficient charge transfer from the dye to TiO_2_.

**Table 2:** Adsorption Energy (*E*_ads_) in eV, Change in LUMO Energy before and after the Adsorption in eV, λ_max_ in nm of the Designed Dyes/TiO_2_ Complexes, and Exciton Binding Energy (*E*_b_) in eV^a^

	*E*_ads_		^b^Δ*E*_LUMO_		λ_max_		Δ*G*_*i*nj_ (eV)		*E*_b_	
name	BA	CA	BA	CA	BA	CA	BA	CA	BA	CA
AJ9	–0.92	–0.72	0.05	0.11	942.56	943.81	–0.33	–0.20	0.15	0.09
AJ10	–0.61	–0.50	0.01	0.09	882.04	931.05	–0.28	–0.17	0.14	0.05
AJ11	–0.60	–0.78	0.03	0.26	904.02	921.00	–0.23	–0.14	0.11	0.03
AJ12	–0.39	–0.33	–0.01	0.14	970.71	965.40	–0.28	–0.19	0.14	0.10
AJ13	–0.44	–0.29	0.03	0.13	954.50	958.67	–0.22	–0.16	0.17	0.08
AJ14	–0.43	–0.13	0.03	0.11	957.18	952.74	–0.23	–0.14	0.14	0.06
ref	–1.99		0.59		473.89		–1.51		0.02	

a All of the calculations are performed in the THF solvent. The rest of the dyes are included in Table S3.

b Δ*E*_LUMO_ = *E*_LUMO_^isolated dye^ – *E*_LUMO_^dye@TiO_2_^.

The injection energy can be calculated as^66^ Δ*G*_*i*nj_ = *E*_dye*_ – *E*_CB_, where *E*_CB_ is the reduction potential of the conduction band (CB) of the TiO_2_ cluster. *E*_CB_ corresponds to the LUMO energy (−2.75 eV) of the cluster. The oxidation potential of the excitation of the dye (*E*_dye*_) can be computed by subtracting the vertical transition energy (λ_max_) from the redox potential of the ground state of the dye (*E*_dye_), whereas *E*_dye_ = −*E*_HOMO_, according to Koopmans’ theorem. All of the designed dyes show the negative values of Δ*G*_inj_ (Tables 2 and S4), implying that the dyes’ excited states lie above the CB edge of TiO_2_ with a compelling charge transfer excitation character.^67^

### 3.5 UV–Vis Absorption Properties of TiO_2_-Bound Dyes

The characteristics of electronic transitions corresponding to the low-lying excited states have been explored within the TDDFT framework using the OT-ωB97XD/6-31G­(d,p) level of theory to elucidate the influence of different π-conjugations and acceptor groups. The simulated absorption spectra of the AJ dyes@TiO_2_ cluster are presented in Figures 4 and S7. All of the AJ dyes (except AJ12-CA) exhibit a red-shifted behavior compared to their free form. The maximum absorption peak of the bound AJ6 dyes is located at 1057 and 1062 nm for the BA and CA acceptor groups, respectively. The red-shifted behavior indicates that the strong binding has been achieved via the −COOH group with TiO_2_, which is conducive to the increased PCE of the DSC.

The FMO distributions of 40 AJ dyes@TiO_2_ are shown in Figure S9. The HOMO of all of the dyes is primarily distributed on the part of the dyes and in a similar position as the HOMOs of single dyes, while LUMOs are populated either in TiO_2_ (AJ1, AJ2, AJ4, and AJ7) or in π-bridges. For the latter case, the electron density moves sequentially like D (HOMO) to π to A (LUMO) and TiO_2_ (LUMO+1). For the former group, such electron delocalization has sufficient driving force to promote the transfer and injection of electrons to TiO_2_ and its CB, respectively.

### 3.6 Excited-State Dynamics

The excited-state lifetime (τ) of the dye is a critical factor in evaluating the efficiency of charge transfer via injection of the excited-state electron. A dye with a longer lifetime in the excited state is expected to be more facile for charge transfer. Also, the lifetime (τ) of the first excited singlet electronic state (*S*_1_) is crucial to modulate the charge transfer phenomenon efficiently. This can be estimated by the relationship from Chaitanya et al.^68^ τ = 1.499 cm^–2^ *s*/(*fE* ^2^); where *E* (cm^–1^) is the excitation energy for *S*_1_ geometry and *f* is the oscillator strength for the respective state. The dye with a more extended lifetime in the first excited state is predicted to transfer charge efficiently.^69,70^ The computed excited-state lifetime of the AJ dyes, along with the MS5 dye, is presented in Figure 5. It is noted that most of the dyes with the BA acceptor group showed the longest excited-state lifetime compared to the CA group. AJ3-BA/CA, AJ6, and AJ9-AJ11 show higher τ compared to MS5, while the maximum τ is for AJ6-CA with 58 ns. The presence of the longest τ (or the slowest injection rate) is thought to be beneficial to the overall efficiency of the DSC due to the expected minimized charge recombination afterward.^71^ These findings suggest that a lower aggregation is expected from those dyes due to slow charge transfer dynamics.^72^

**Figure 5:**
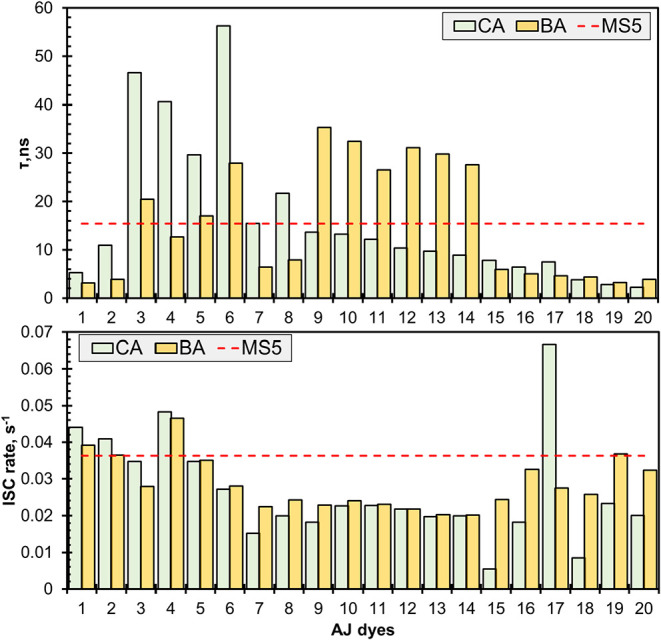
Intersystem crossing (left) and excited-state lifetime (right) are given along with the values of reference dyes (red dashed lines).

Another important excited-state property is the rate of intersystem crossing (ISC), a nonradiative pathway from the singlet to triplet state, of the excited dyes, which affects the overall performance of DSCs. The ISC rate can be computed based on the equation of semiclassical Marcus’ theory^73^ (eq 1), based on the total reorganization energy, λ_Total_, (the sum of the inner (λ_in_) and outer (λ_out_) energies due to the solvent).

1 $$k_{\mathrm{ET}}=\frac{1}{\sqrt{\lambda_{\mathrm{Total}}}}\sqrt{\frac{\pi }{\hbar^{2}k_{B}T}}\left|V^{2}\right|\exp\left\{-\frac{\lambda_{\mathrm{Total}}}{{4k}_{B}T}\right\}$$

On the right-hand side of the equation, *k*_B_ (Boltzmann constant) and ℏ (Planks’ constant) are constants. |*V* ^2^| is the coupling term. Samanta et al.^74^ showed that the spin–orbit coupling and the energy difference between the S_1_ and T_1_ states yields the similar values of the ISC rate. In this work, we compute the rate of ISC using the relation, λ_in_^ISC^ = *E*_s_^T^ – *E*_T_^T^, where λ_in_^ISC^, *E*_s_^T^, and *E*_T_^T^ are the inner reorganization energy, the energy of the triplet state at singlet-state geometry, and the energy of the triplet state at triplet-state geometry.^74,75^ The computed rate of ISC is presented in Figure 6 along with the reference dye. A lower value of λ_in_^ISC^ is imperative for efficient electron injection to compete with other de-excitation processes. Most of the AJ dyes show a lower ISC than the MS5 dye (0.036), while the lowest ISC is for AJ15-CA (0.005). Based on the acceptor groups, there is no correlation with the ISC. However, if we closely examine the symmetry of the conjugation fragments, the asymmetric motif shows a relatively lower ISC. Unlike metal complexes, asymmetric molecules with light atoms show a very slow ISC.^76^ These findings correlate with the *E*_gap_ of the dye’s ground-state properties and excited-state lifetime.

**Figure 6:**
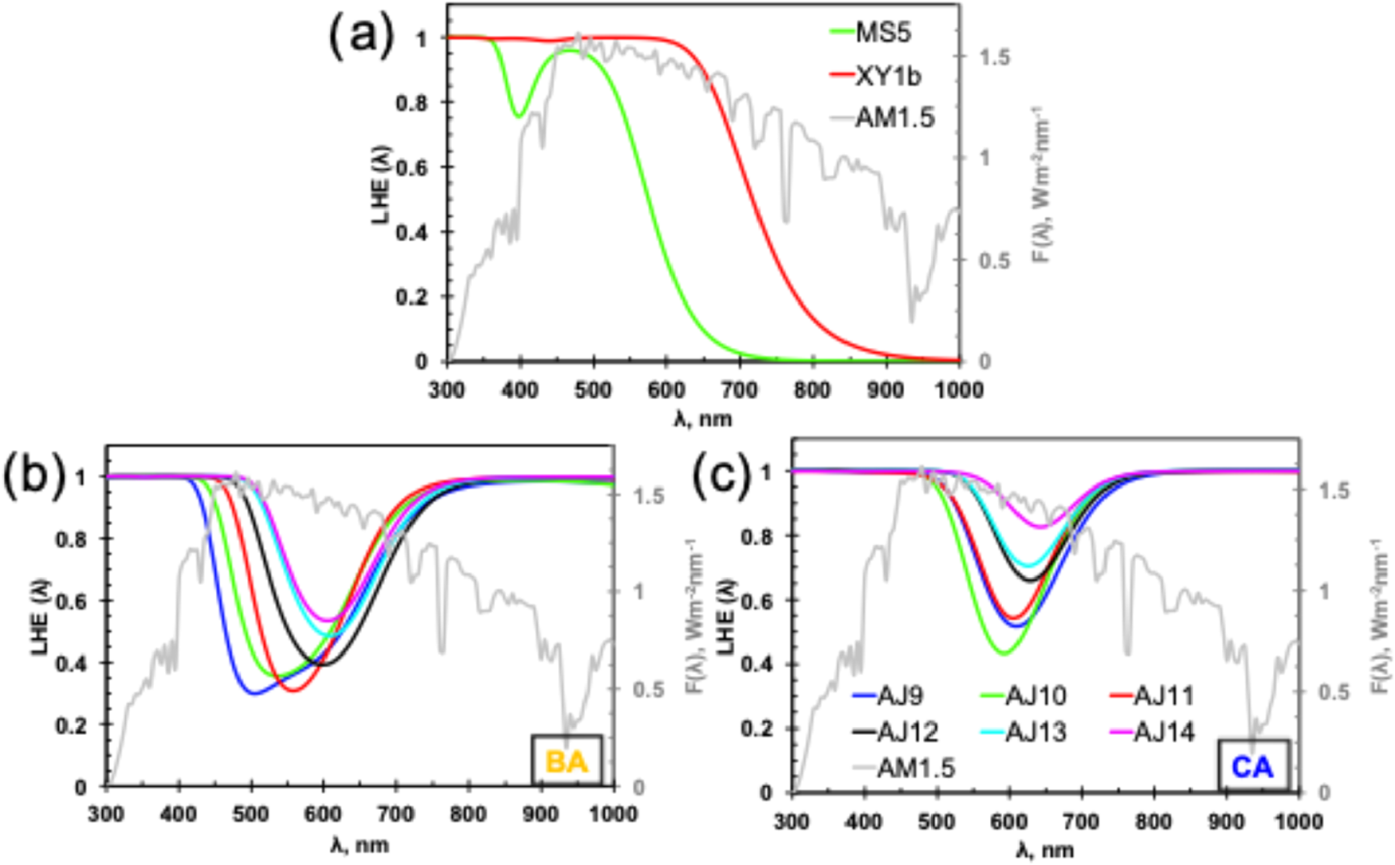
Calculated LHE (λ) curves of the (a) reference MS5 and XY1b dyes and (b, c) designed dyes with the BBT fragment along with the solar spectrum (AM1.5).

To understand the CT rate, we compute λ_Total_ by defining and taking the sum of the electron (λ_e_) and hole (λ_h_) reorganization energy. The calculation is described in the Supporting Information Note 2.

The computed values are listed in Table 3. The computed value of λ_Total_ for the designed dyes is lower than that of the MS5 dye (0.56) and the lowest value is 0.12 for AJ6-BA. The rate of CT increases with decreasing λ_Total_, which complies with the ISC findings.

**Table 3:** Estimated *J*_SC_^max^ (mA cm^–2^), *V*_OC_ (in eV), Total Reorganization Energies (λ_total_ in eV), μ_normal_, Excited-State Oxidation Potential, and *e*_grad_^–^ of the Isolated AJ Dyes with MS5 in the THF Solvent^a^

	*J*_SC_^max^ (mA cm^–2^)		*V*_OC_ (eV)		λ_total_		μ_normal_		ESOP		*e*_grad_^–^	
name	BA	CA	BA	CA	BA	CA	BA	CA	BA	CA	BA	CA
AJ1	30.15	33.61	1.98	1.31	0.52	0.61	7.56	10.51	–2.04	–2.3	2.86	2.62
AJ2	29.07	33.82	1.90	1.28	0.47	0.61	10.86	13.63	–2.11	–2.38	2.79	2.53
AJ3	41.49	45.78	0.50	0.39	0.37	0.41	15.23	18.05	–3.32	–3.4	1.56	1.54
AJ4	26.92	31.87	1.07	0.95	0.47	0.5	13.16	15.13	–2.78	–2.84	2.13	2.09
AJ5	38.78	44.62	0.63	0.55	0.43	0.48	15.17	18.28	–3.26	–3.36	1.69	1.66
AJ6	38.61	49.11	0.32	0.26	0.12	0.23	16.04	16.78	–3.32	–3.42	1.48	1.40
AJ7	30.59	34.35	1.19	1.04	0.43	0.47	10.84	14.79	–2.54	–2.60	2.41	2.36
AJ8	29.77	33.80	0.98	0.87	0.39	0.44	14.23	12.08	–2.64	–2.70	2.30	2.26
AJ9	58.56	71.24	0.48	0.29	0.36	0.45	15.32	27.77	–3.43	–3.57	1.37	1.30
AJ10	57.68	68.61	0.42	0.22	0.32	0.34	8.20	23.8	–3.52	–3.60	1.42	1.35
AJ11	63.15	69.05	0.34	0.16	0.30	0.36	14.09	30.43	–3.57	–3.63	1.39	1.34
AJ12	70.85	73.82	0.42	0.29	0.36	0.28	11.48	25.97	–3.48	–3.55	1.28	1.28
AJ13	70.57	73.11	0.39	0.24	0.34	0.29	15.78	26.63	–3.57	–3.63	1.29	1.30
AJ14	70.51	73.71	0.36	0.19	0.30	0.31	16.57	25.91	–3.57	–3.65	1.31	1.30
AJ15	37.00	40.37	1.02	0.75	0.42	0.48	8.22	20.52	–2.56	–2.67	2.40	2.25
AJ16	35.84	36.33	1.00	0.94	0.48	0.52	5.56	16.08	–2.58	–2.57	2.47	2.40
AJ17	38.34	40.71	0.88	0.56	0.44	0.53	12.41	21.41	–2.67	–2.74	2.40	2.24
AJ18	40.50	41.99	0.97	0.75	0.41	0.44	12.75	21.11	–2.58	–2.59	2.38	2.31
AJ19	40.63	42.11	0.99	0.73	0.47	0.49	14.31	21.99	–2.64	–2.65	2.41	2.33
AJ20	40.69	42.70	0.90	0.60	0.42	0.51	14.22	20.88	–2.65	–2.69	2.40	2.30
MS5	50.83		1.53		0.56		15.01		–4.93		2.66	

a e_grad_^–^ = |ESOP – GSOP|.

### 3.7 Simulation of Photovoltaic Parameters of Dye Molecules

The overall efficiency of photo-to-electron conversion in DSCs is determined by the relation^77^ described in eq 2, which relates the short-circuit photocurrent density (*J*_SC_), open-circuit photovoltage (*V*_OC_), fill factor (FF), and incident solar power on the cell using eq 2.

2 $$\mathrm{PCE}=\frac{J_{\mathrm{SC}}\times V_{\mathrm{OC}}\times \mathrm{FF}}{P_{\mathrm{IN}}}\times 100\%$$

#### 3.7.1 Short-Circuit Current Density

The evaluation of the proposed AJ dyes’ photovoltaic parameters was undertaken to comprehend the variations concerning the theoretical performances of MS5 in DSCs. The *J*_SC_ of DSCs is mainly linked to the five factors, including the light-harvesting efficiency (LHE), electron injection efficiency (Φ_inj_), electron collection efficiency (η_col_), dye regeneration efficiency (η_reg_), and photon flux φ­(λ) of incident light at AM1.5 G and can be expressed as

2a $$J_{\mathrm{SC}}=e\int_{\lambda }\varphi \left(\lambda \right)\mathrm{LHE}\left(\lambda \right)\Phi_{\mathrm{inj}}\eta_{\mathrm{col}}\eta_{\mathrm{reg}}d\lambda$$

2b $$J_{\mathrm{SC}}=e\int_{\lambda }\varphi \left(\lambda \right)\mathrm{LHE}\left(\lambda \right)d\lambda$$

where the LHE of the dye molecules can be obtained by the equation

3 $$\mathrm{LHE}=1-10^{-\Gamma \sigma \left(\lambda \right)}=1-10^{-f}$$

η_col_ is a constant for the same DSC as it only depends on the architecture of the device. η_reg_ and Φ_inj_ are close to unity as the incident photon-to-efficiency is near unity under AM1.5 sun illumination.^78^ As a result, the enhancement of *J*_SC_ should focus on improving the LHE­(λ).^79^ Here, Γ is the loading amount of dyes on the TiO_2_ surface (mol cm^–2^) and σ is the absorption cross-section (cm^2^ mol^–1^), which is obtained by multiplying ϵ (M^–1^ cm^–1^) by 1000 cm^3^ L^–1^. Referring to the experimental value of MS5 in practice, all of the designed dye loading amounts were set to 6.35 × 10^–8^ mol cm^–2^.^10,13^ Combining the Γ values with the simulated data in UV–vis absorption spectra, the derived LHE curves of the AJ dyes at different absorption wavelengths are depicted in Figures 6 and S10. Computed maximum *J*_SC_ (*J*_SC_^max^) values of the AJ dyes are listed in Table 3. We used two different approaches to compute the LHE and thus *J*_SC_^max^. In both cases, the computed *J*_SC_^max^ is larger for the AJ9-AJ14 dyes, aligning with the UV–vis results. Also, results revealed that the *J*_SC_^max^ is higher for the AJ dyes with CA. In Figure 6, we can see that the LHE value of MS5 formed a valley around 400 nm, while XY1b^13^ maintained a plateau up to 700 nm, which are complementary to each other. Our new designed dye also can be used with XY1b to harvest photons in the NIR. Results from the simulated LHE curves are aligned with the discussion in Section 3.3.

#### 3.7.2 Open-Circuit Voltage

The open-circuit photovoltage (*V*_OC_) that can be expressed^80^ by eq 6 depends on the recombination of the dye with the electrolyte. This type of recombination could provide an insufficient blocking effect due to the dyes’ relatively low adsorption density.^81^ Thus, to enhance the effect, the shape and structures of the dyes need to be optimized. Ning et al. suggested that upshifting of the sensitizer E_LUMO_ will increase the *V*_OC_ and reduce the charge recombination.^82^

4 $$V_{\mathrm{OC}}=E_{\mathrm{LUMO}}-E_{\mathrm{CB}}^{\mathrm{Ti}O_{2}}$$

Besides, *V*_OC_ can be estimated by the following relation in terms of the conduction band edge (E_CB_) of the semiconductor:^83^

5 $$V_{\mathrm{OC}}=\frac{E_{\mathrm{CB}}}{q}+\frac{\mathrm{kT}}{q}\ln\left(\frac{n_{c}}{N_{\mathrm{CB}}}\right)-\frac{E_{\mathrm{redox}}}{q}$$

where *E*_redox_ is the electrolyte Fermi level, *kT* is the thermal energy, *q* is the unit charge, *n*_c_ is the number of electrons in the conduction band, and *N*_CB_ is the accessible density of CB states. After dye adsorption on the semiconductor, *E*_CB_ can be expressed as the absolute value of the dipole moment of the individual dye molecule perpendicular to the surface of the semiconductor surface (μ_normal_) as given by eq 5 ^84^

6 $$\Delta \mathrm{CB}=-\frac{q\mu_{\mathrm{normal}}\gamma }{\epsilon_{0}\epsilon }$$

where *q* is the electron charge, γ is the dye’s surface concentration, and ϵ_0_ and ϵ are the permittivity of vacuum and the dielectric constant of the organic monolayer, respectively.

*V*_OC_ is one of the salient features of DSCs, which can be estimated by eq 8. The shift of the CB of TiO_2_ after dye adsorption can be determined qualitatively. Besides, shifting of the CB is related to the μ_normal_ of the dyes^84^ and the synergistic effect of two factors will lead to an increase in *V*_OC_, for a defined redox shuttle in DSCs.^85,86^ The larger the μ_normal_ of the adsorbed dye molecules, the higher is the *V*_OC_ value. Here, we estimated the *V*_OC_ of the designed dyes by quantifying the μ_normal_ values of the adsorbed dyes. We have computed the μ_normal_ of the dyes with the solvent effect based on the protocol suggested by Grätzel et al.,^87^ considering the *C*_*2*_ axis of the carboxylate in the dye parallel to the *x*-axis and the TiO_2_ surface parallel to the *yz*-plane. This parameter generally exhibits higher values for CA acceptors than for BA acceptors. The larger μ_normal_ values suggest that AJ dyes with the CA acceptor group should be characterized by the increased *V*_OC_ and hence the enhanced PCE of DSCs. Often, the theoretical prediction of *V*_OC_ provides an upper limit, as it does not consider the conditions of an actual device, such as nonradiative recombination, charge carrier collection losses, interfacial recombination, and resistance losses.^88,89^

### 3.8 Photostability of the Designed Dyes

Photostability is one of the factors that affect the duration of DSC devices. To screen the photostability of the designed dyes, we have implemented a very fast and robust screening method proposed by De Angelis and co-workers,^43,90^ which depends on the aligned excited-state energy or excited-state oxidation potential (ESOP). The aligned level of ESOP is the vital factor that governs the stability of the photoexcited dyes. The value of ESOP was computed by the free energy differences of neutral and oxidized dyes in the excited state at their equilibrium geometry of the neutral species. ESOP may be approximated by ESOP ≅ GSOP – *E*_0–0_, where *E*_0–0_ is the energy difference between optimized excited and ground states, while GSOP is the ground-state oxidation potential. Due to the large systems considered here, the approximation *E*_0–0_ ≈ vertical excitation energy is applied. We estimated GSOP by the approximation *GSOP* = [*E*_neutral_ – *E*_cation_]_GS_; finally, ESOP was computed by adding the absorption energy (*E*_abs_) to GSOP. The gradient of electron injection (e_grad_^–^) to the CB of TiO_2_ can be estimated by taking differences between ESOP and GSOP. The values of ESOP and e_grad_^–^ are tabulated in Table 3. Tabulated data show that all CA-based AJ dyes have lower ESOP than BA. Thus, the e_grad_^–^ is also lower, which facilitates the photostability of the AJ dyes after the fast electron injection to the CB of TiO_2_. Among the CA-based AJ dyes, AJ6 and AJ9-AJ14 dyes exhibit the lowest uphill e_grad_^–^. Charge accumulation at the interface favors the continuous electron injection from the dye to the surface, forming a built-in electric field.^70^ These results support the conclusion of the previous discussions concerning the excited-state lifetime and ICT discussions.

## 4 Conclusion

We systematically designed and investigated the effect of π-spacers and acceptors (benzoic and cyanoacrylic acid) on the photophysical properties of the BTPA-based dye sensitizers via DFT and TDDFT calculations. We also elucidate the interfacial behavior of the designed dyes using the (TiO_2_)_16_ cluster model. Our calculations revealed that the AJ dyes with BBT fragments show more promising HOMO–LUMO gaps than iso-BBT dyes in accessing the NIR-region wavelength light. The asymmetry of the π-spacers is the decisive factor in lowering the gap and thus the bathochromic shift of the absorption peak. The asymmetric motif shows a relatively lower ISC and a higher excited-state lifetime. Dyes with BBT fragments like AJ6 and AJ9 to AJ14 show better photostability compared to the reference dye MS5. The spectral compatibility of AJ dyes with MS5 makes them potential candidates for cosensitized DSC devices. Our estimated photovoltaic parameters found that the *J*_SC_ of AJ9-AJ14 (BA and CA) and the *V*_OC_ of AJ1-BA and AJ2-BA dyes are higher than those of MS5. We found that the dyes with CA showed better photophysical properties. The results proved that rational molecular design provides a valuable reference for synthesizing dyes with higher efficiency. In summary, different strategies for the molecular design of organic dyes result in varying photovoltaic properties. Computational studies help to understand how structural modifications affect the dye performance in solar cells.

## Supplementary Material



## Notes

Detailed benchmarking of DFT/TDDFT functionals including ω tuning, electron density map of the HOMO and LUMO of isolated dyes and dye/TiO_2_, UV–vis absorption spectra, structural attributes, and excited-state properties of the dyes (PDF)
