# Supplementary material for: Role of π‑Spacers and Acceptors in Regulating the Photophysical Properties of BTPA Donor-Based Dyes: First-Principles Approach
Source: J Phys Chem A. 2025 Nov 20;129(48):11104–16. doi: 10.1021/acs.jpca.5c05194 (PMC12683642; doi:10.1021/acs.jpca.5c05194)
Supplement: Supplementary file 1 [file jp5c05194_si_001.pdf]

Supporting Information for Publication

Role of π-spacers and Acceptors in Regulating the Photophysical Properties of BTPA Donor-based Dyes: First-principles Approach

Juganta K. Roy*, Asmita Adhikari, Tyler Lafferty

Clean Energy Materials Modeling Laboratory, Department of Chemistry and Physics, West Texas A&M University, Canyon, TX 79016, USA

*Corresponding author:

Prof. Juganta K. Roy, Phone: +1 806-651-4266; fax: +1806-651-2928; E-mail: jroy@wtamu.edu

Figure/Table	Name of the Figures/Table	Page no.
Fig. S1	Benchmarking of the DFT functional	S2
Fig. S2	Benchmarking of the TDDFT functional, OT-ωB97XD (OT= optimally tuned)	S3
Fig. S3	Optimized geometry of the designed AJ dyes with acceptor group (a) benzoic acid and (b) cyanoacrylic acid.	S4
Fig. S4	Computed energies of frontier molecular orbitals including HOMO and LUMO.	S5
Fig. S5	Electron density distribution map of HOMO, LUMO and LUMO+1 of AJ dyes: acceptor group benzoic acid (BA)	S6
Fig. S6	Electron density distribution map of HOMO, LUMO and LUMO+1 of AJ dyes: acceptor group cyanoacrylic acid (CA)	S7
Fig. S7	Simulated UV-Vis absorption spectra of the isolated AJ dyes & dyes@TiO_2 cluster	S8-S9
Fig. S8	Optimized geometry of the dye@TiO_2 cluster	S10
Fig. S9	Electron density map like HOMO, LUMO and LUMO+1 of designed dyes@TiO_2 cluster.	S11
Fig. S10	LHE curves (a) and (b) BA and (c) and (d) CA acceptor group	S12
Table S1	Computed dihedral angles of the DFT geometry optimized AJ dyes at PBE0/6-31G(d,p) level of theory in THF solvent.	S13
Table S2	Excited states properties of the isolated designed AJ dyes computed at the TD-OT-ωB97XD/6-31G(d,p) level of theory.	S14-S15
Table S3	Excited states properties of the designed AJ dyes@TiO_2 computed at the TD-OT-ωB97XD/6-31G(d,p) level of theory in THF solvent.	S16-S19
Table S4	Photophysical parameters of isolated dyes and dyes bound to cluster	S20

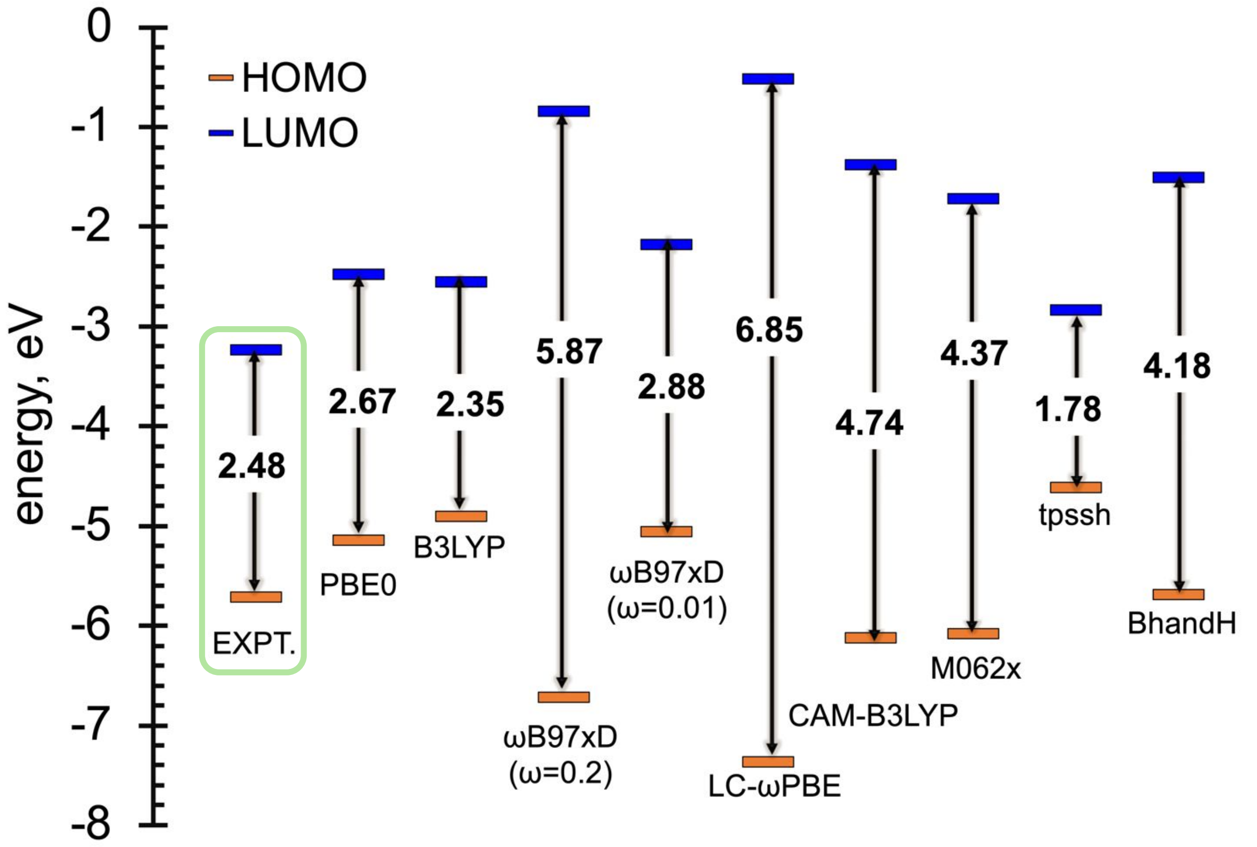

Figure S1. Benchmarking of the functionals for ground state DFT calculations. We compare the computed frontier molecular orbital energies and their gap with the experimental values based on the reference dye, MS5. Our results showed that the PBE0 functional describes the energetics (energy gap of FMOs and their positions) of the reference dye more closely (green box). It was found that PBE0 is better functional than B3LYP to reproduce geometrical features.[1] The experimental HOMO, LUMO and their gap is -5.71 eV, -3.23 eV and 2.48 eV, respectively.

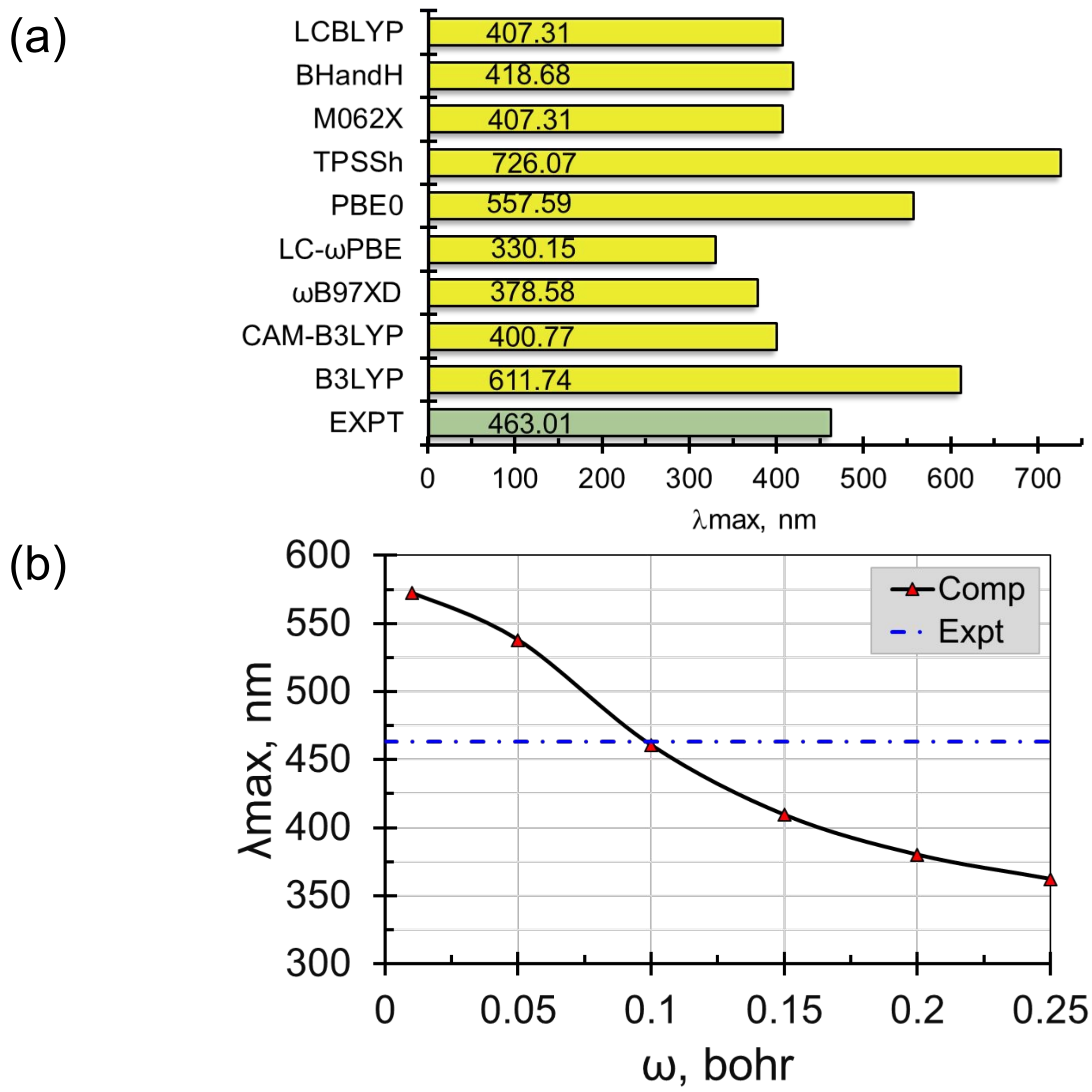

Figure S2. (a) Benchmarking of the functionals for TDDFT calculations. We compare the λ_{max} of the reference dye, MS5, using different functionals. (b) Tuning of range-separated parameter for ωB97XD functional, as it is one of the best functional to reproduce experimental observation for conjugated systems.[2–4] DFT-ωB97XD//OT-ωB97XD/6-31G(d,p) computed λ_{max} shown the best agreement with experimental values for ω=0.1 Bohr. For the rest of the study, we implemented DFT-ωB97XD//OT-ωB97XD/6-31G(d,p) level of theory for all the excited state calculations. All the geometry optimization and single point TDDFT calculations were performed using tetrahydrofuran (THF) solvents. OT indicates optimally tuned.

Note 1: To check the robustness of our TDDFT functionals and its accuracy in predicting the λ_{max} of the AJ dyes, we compared the values of MS5 with its closely related structures like ZS10 (481 nm), ZS11 (484 nm), ZS12 (500 nm)[5], MS4 (468 nm), XY1b (513 nm)[6], SL9 (557 nm), and SL10 (410 nm)[7]. Our results revealed that all the designed dyes have longer λ_{max} than the reported MS5 homologous dyes experimentally found.

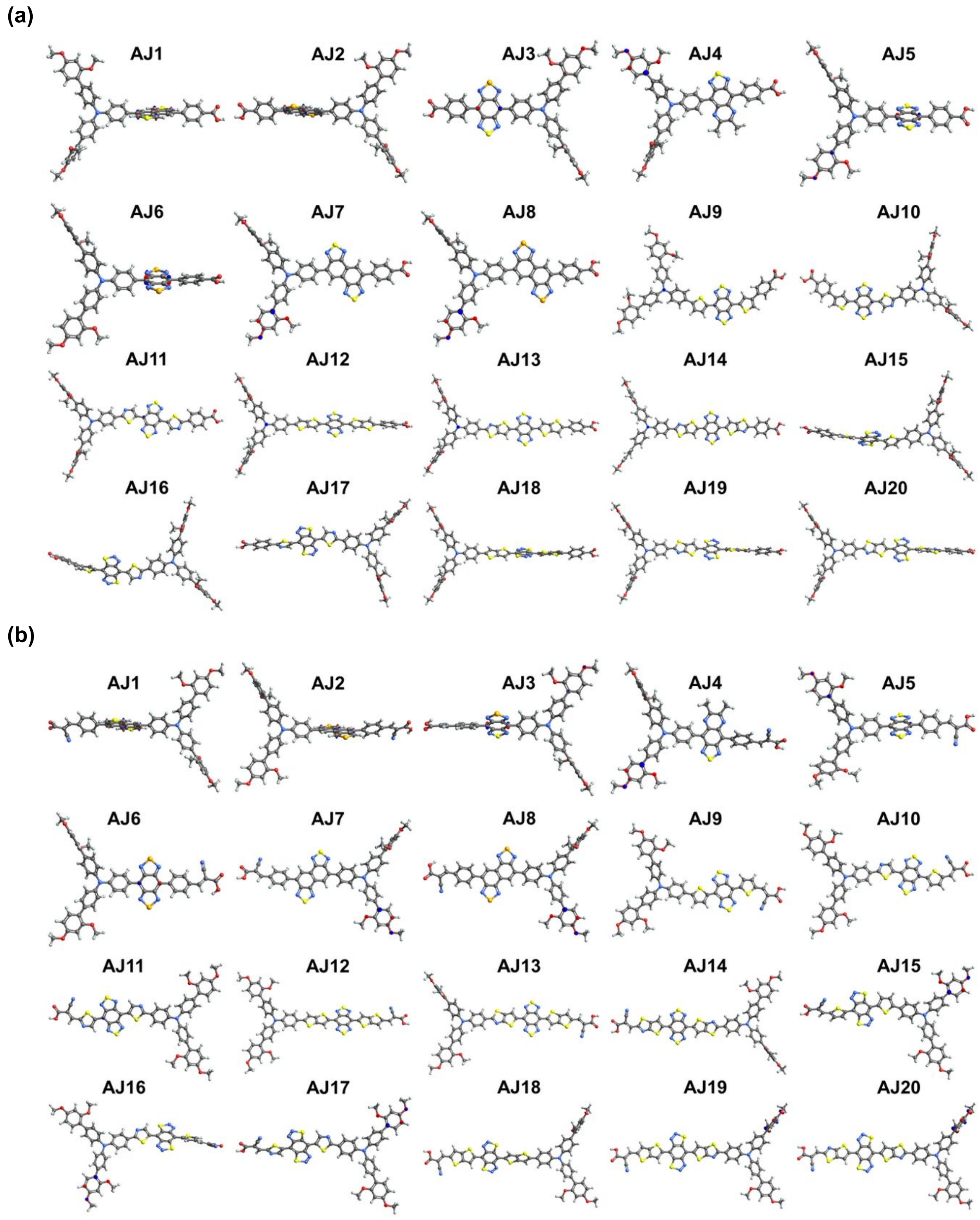

Figure S3. Optimized geometry of the designed AJ dyes with acceptor group (a) benzoic acid and (b) cyanoacrylic acid using DFT PBE0/6-31G (d,p) level of theory in tetrahydrofuran solvent.

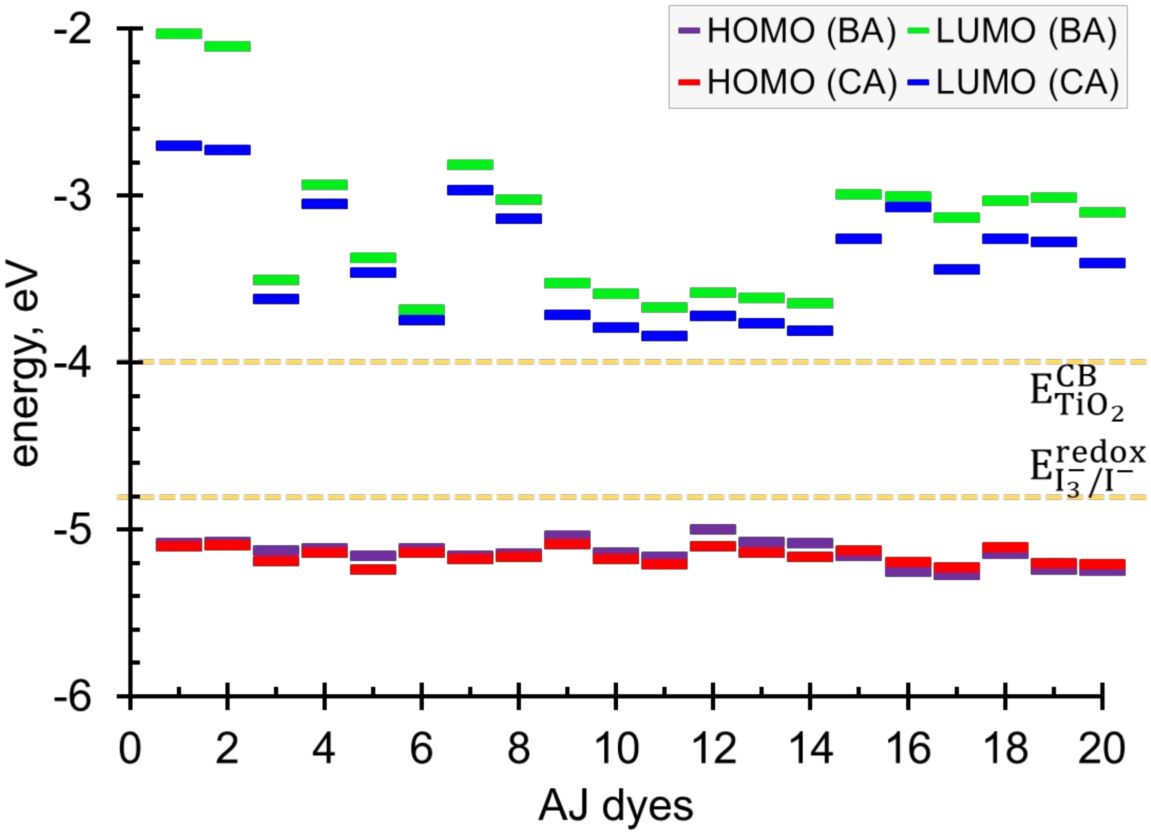

Figure S4. Computed energies of frontier molecular orbitals (HOMO and LUMO) with the conduction band energy of TiO_2 and redox potential of iodide electrolyte. All the calculations performed using DFT PBE0/6-31G (d, p) level of theory in THF solvent.

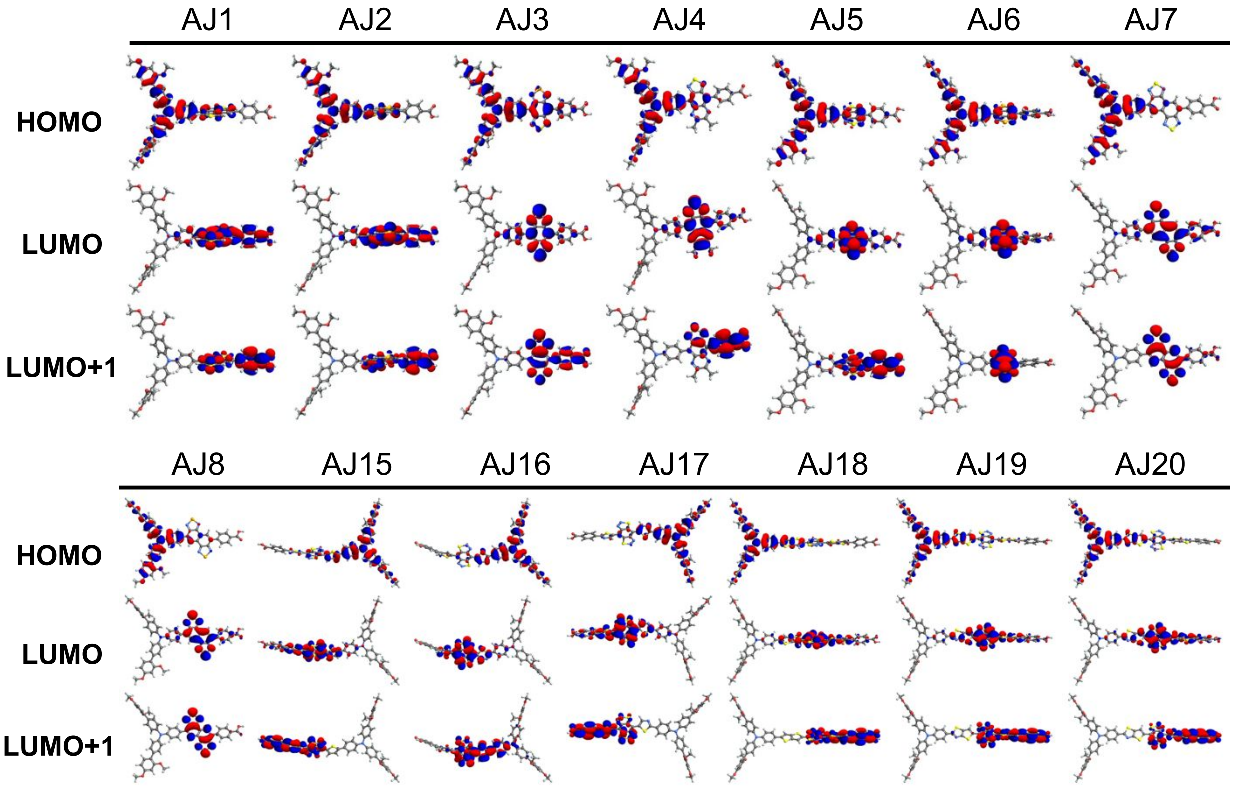

Figure S5. Electron density map of the designed AJ dyes with BA acceptor group.

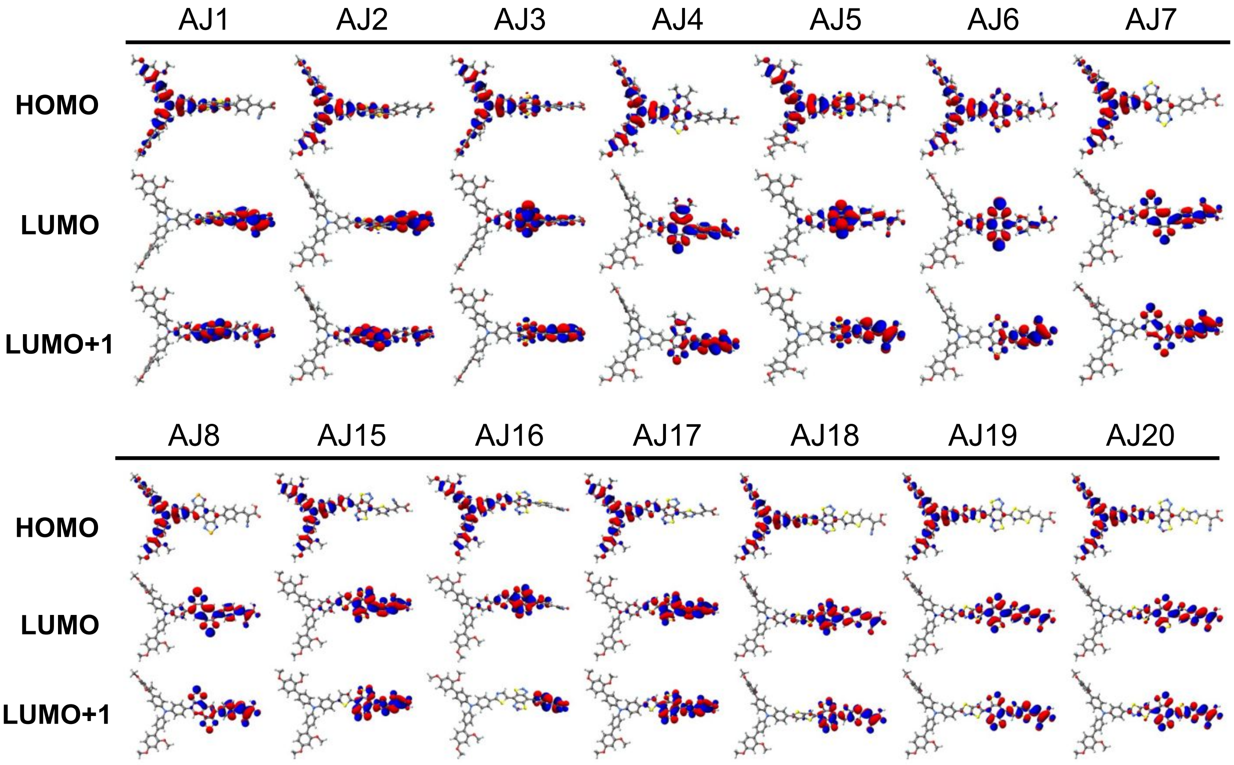

Figure S6. Electron density map of the designed AJ dyes with CA acceptor group.

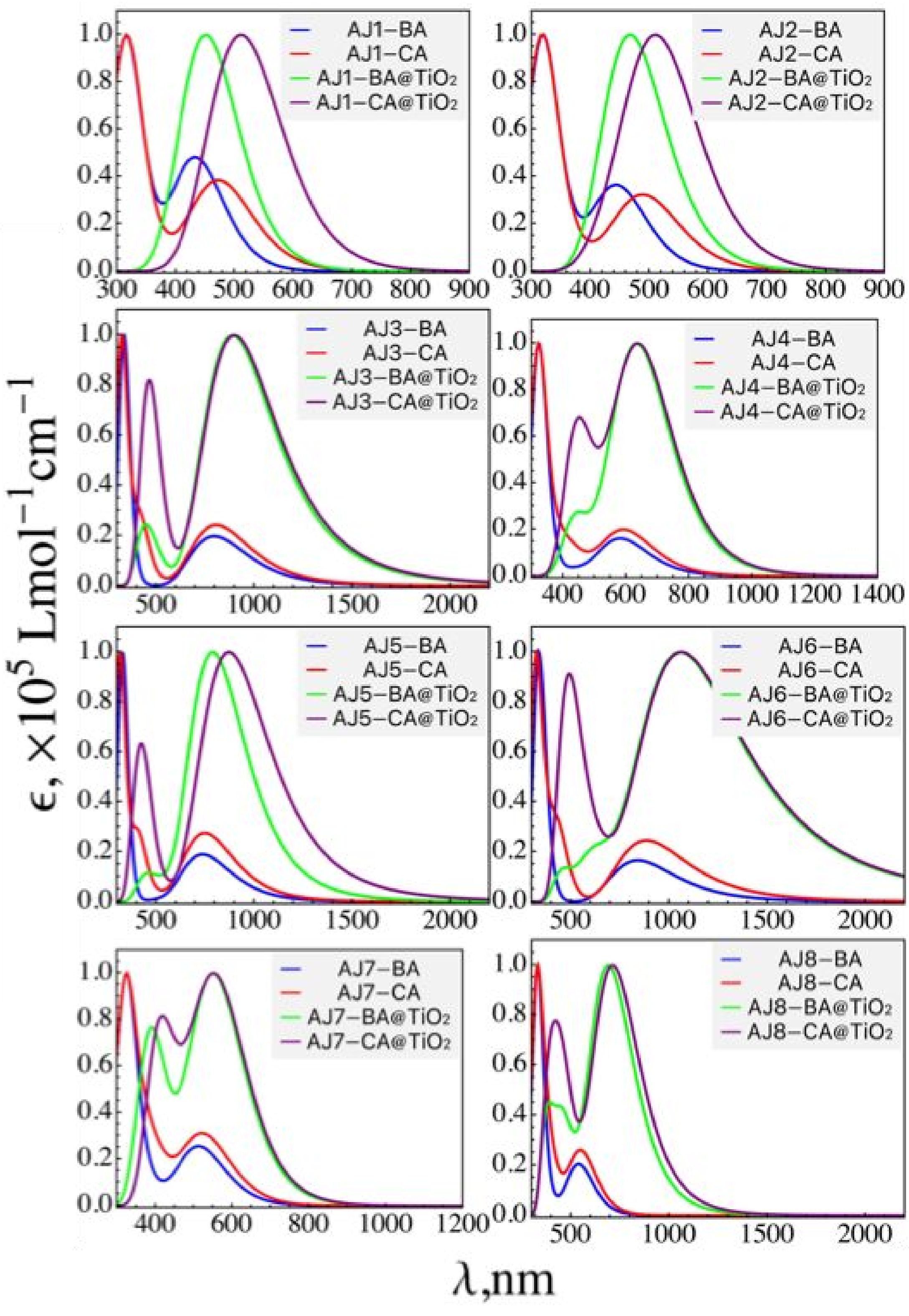
AJ1−BA
AJ1−CA
AJ1−BA@TiO2
AJ1−CA@TiO2
AJ2−BA
AJ2−CA
AJ2−BA@TiO2
AJ2−CA@TiO2
AJ3−BA
AJ3−CA
AJ3−BA@TiO2
AJ3−CA@TiO2
AJ4−BA
AJ4−CA
AJ4−BA@TiO2
AJ4−CA@TiO2
AJ5−BA
AJ5−CA
AJ5−BA@TiO2
AJ5−CA@TiO2
AJ6−BA
AJ6−CA
AJ6−BA@TiO2
AJ6−CA@TiO2
AJ7−BA
AJ7−CA
AJ7−BA@TiO2
AJ7−CA@TiO2
AJ8−BA
AJ8−CA
AJ8−BA@TiO2
AJ8−CA@TiO2
ϵ, ×10^5 Lmol^−1cm^−1
λ,nm

Continution of Figure S7

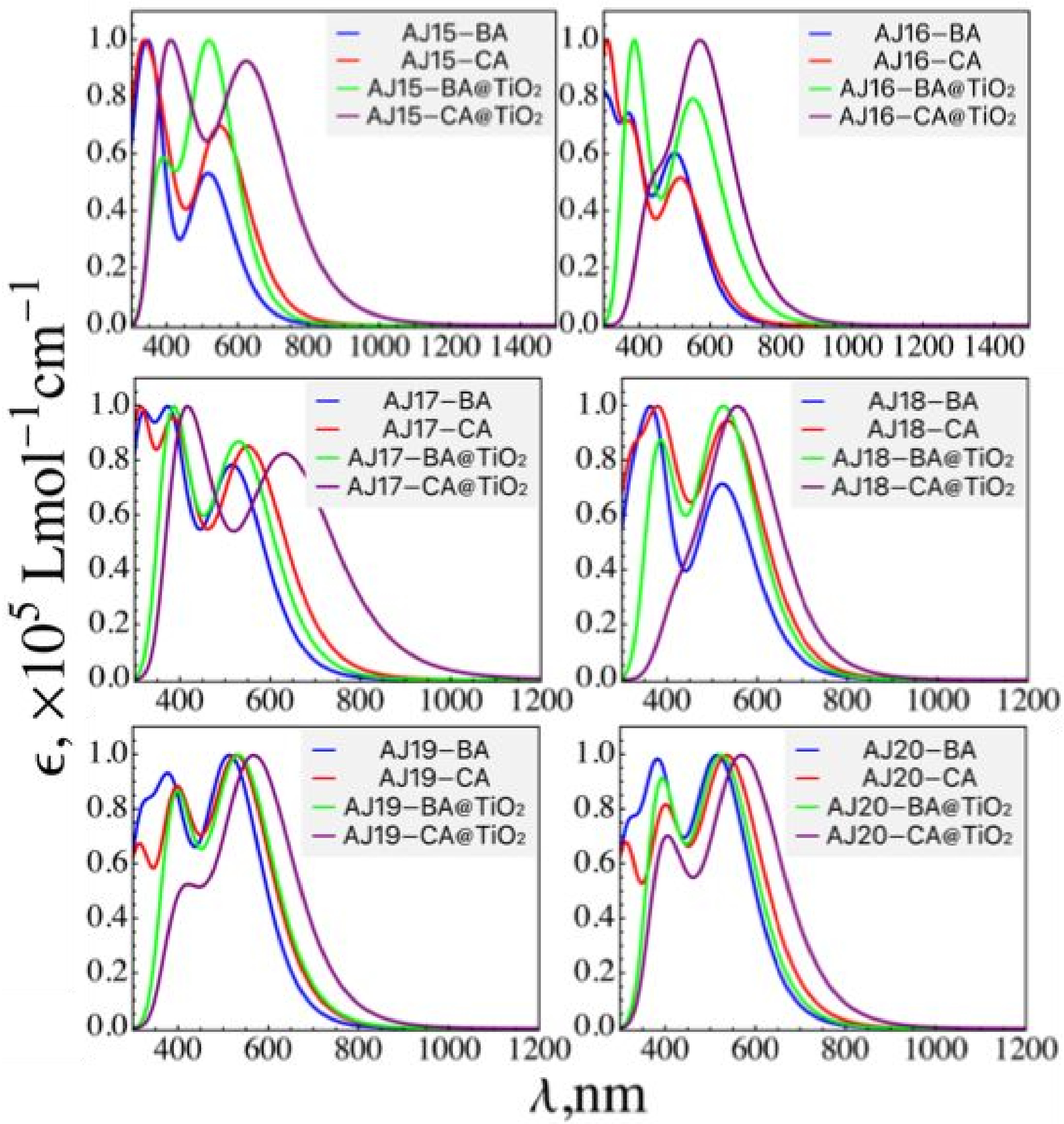

Figure S7. Simulated UV-Vis absorption spectra of the isolated AJ dyes and dye@TiO_2 with benzoic acid and cyanoacrylic acid in THF solvent simulated at the TDDFT/CPCM/OT-ωB97XD/6-31G(d,p) level of theory. For Se and Ti LANL2DZ basis set was used.

(a)

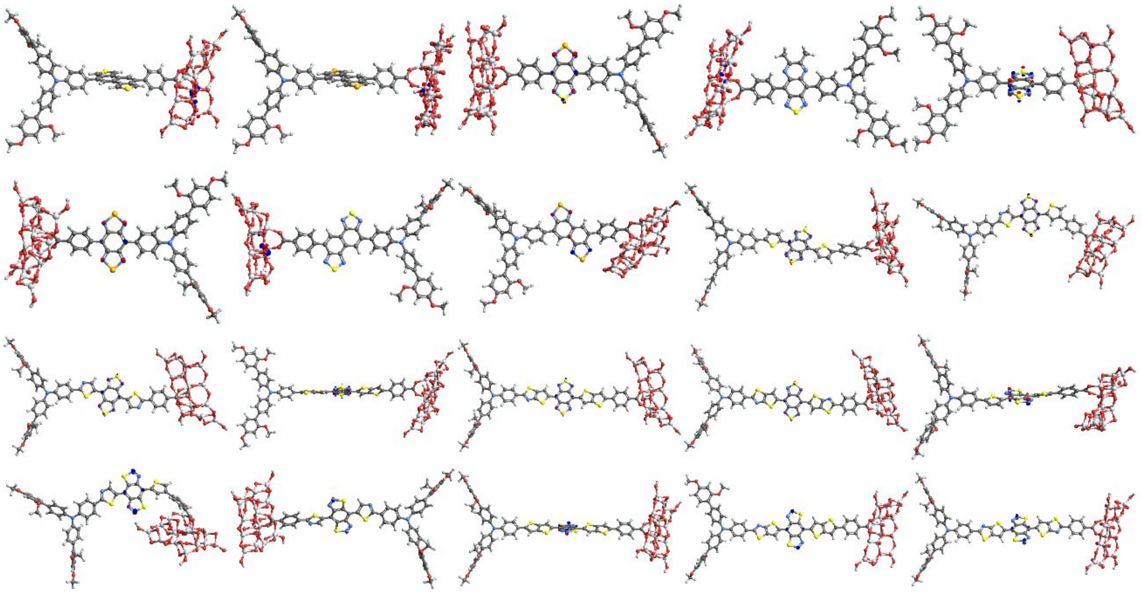

(b)

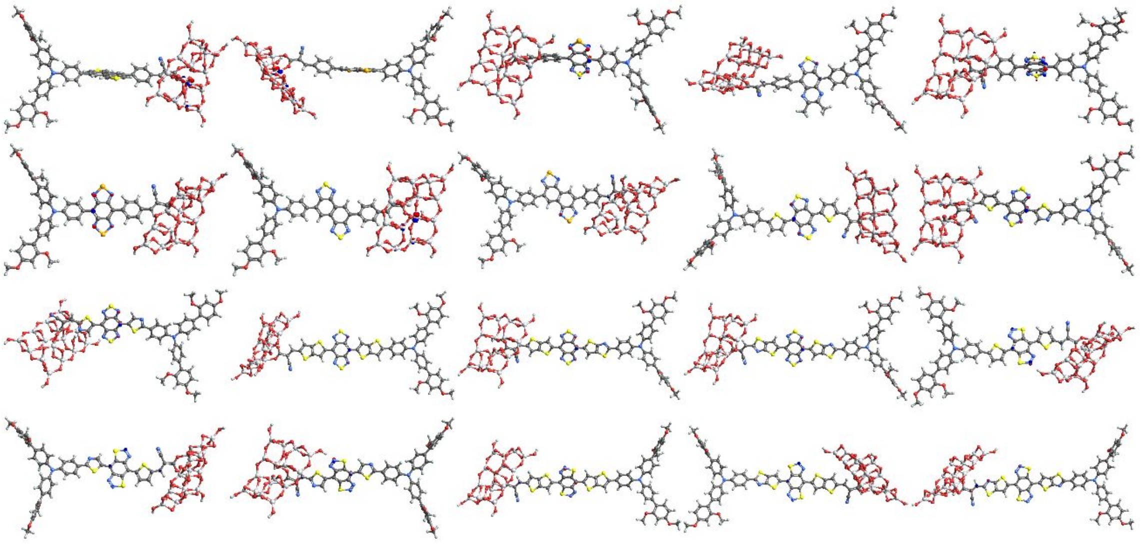

Figure S8. Optimized geometry of the designed TiO_2 bounded AJ dyes with acceptor group (a) benzoic acid and (b) cyanoacrylic acid using DFT PBE0/6-31G(d,p) level of theory in tetrahydrofuran solvent.

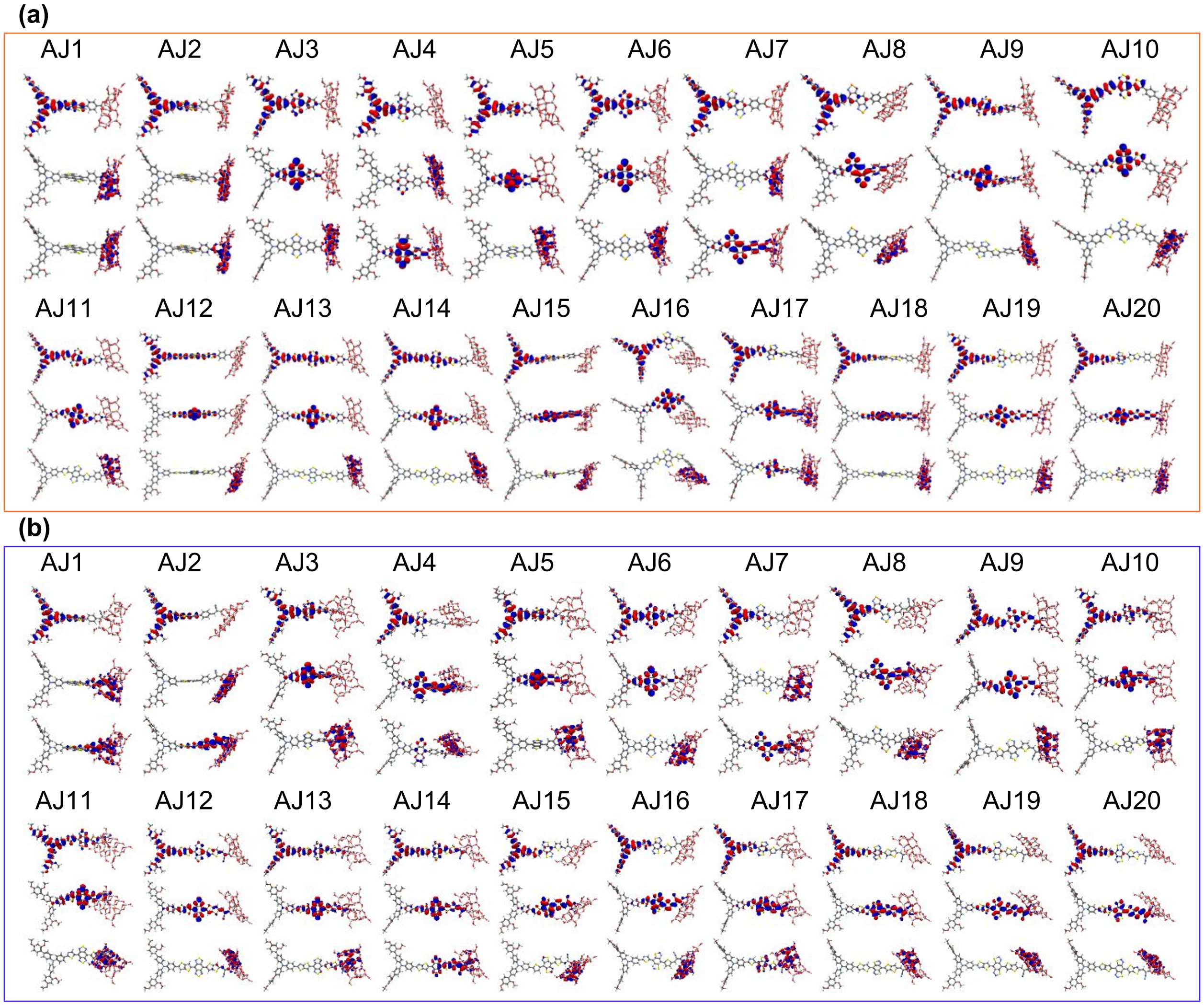

Figure S9. Electron density map like HOMO, LUMO and LUMO+1 of the frontier orbitals of designed dyes@TiO_2 cluster with benzoic acid, BA, acceptor group (a) and cyanoacrylic acid, CA, acceptor group (b).

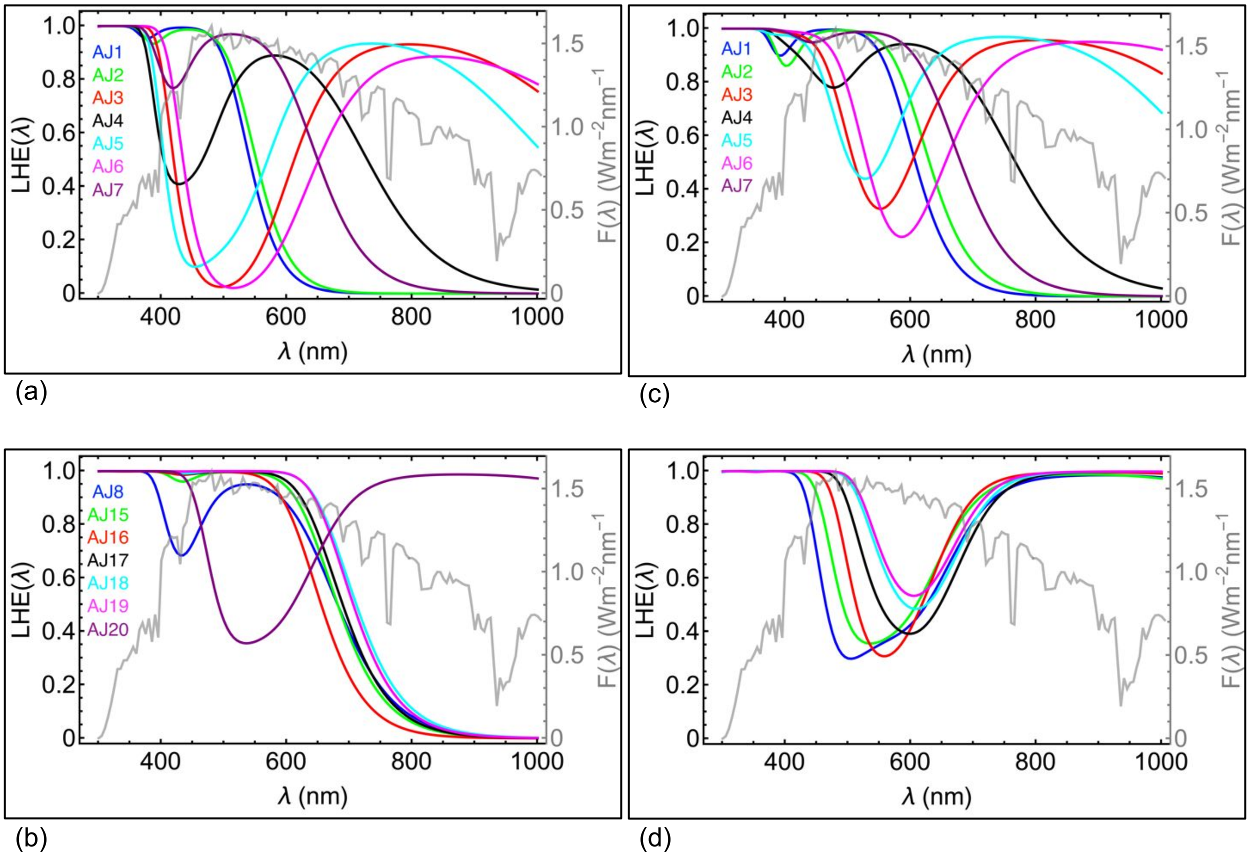

Figure S10. Calculated LHE (λ) curves of the BA acceptor group (a and b) and CA acceptor group (c and d) along with solar spectrum (AM 1.5) in gray color.

Note 2: Calculation of reorganization energy[8]

$$\lambda_{Total} = \lambda_h + \lambda_e$$

$$\lambda_h = \left(E_0^+ - E_+^+\right) + (E_+^0 - E_0^0)$$

$$\lambda_e = \left(E_0^- - E_-^-\right) + (E_-^0 - E_0^0)$$

Where, E_0^+ (E_0^-) denotes the energy of cation (anion) calculated from the optimized structure of the neutral molecule; E_+^+ (E_-^-) is the energy of cation (anion) calculated from the optimized cation (anion) geometry; E_+^0 (E_-^0) is the energy of neutral molecule computed from the cationic (anionic) state; and E_0^0 is the energy of a neutral molecule at the ground state.

Table S1: Computed dihedral angles of the DFT geometry optimized AJ dyes at PBE0/6-31G(d,p) level of theory in THF solvent.

Name	β_1 (deg.)		β_2 (deg.)	
	BA	CA	BA	CA
**AJ1**	135.24	135.02	135.03	135.45
**AJ2**	134.05	132.61	131.83	134.48
**AJ3**	144.25	144.07	140.68	143.62
**AJ4**	134.73	136.75	132.50	133.90
**AJ5**	144.35	144.73	141.10	143.81
**AJ6**	142.18	144.67	139.37	143.14
**AJ7**	146.27	146.58	142.17	145.56
**AJ8**	145.00	146.11	142.65	145.07
**AJ9**	162.29	165.60	152.30	179.94
**AJ10**	177.51	177.08	152.46	179.83
**AJ11**	176.48	177.56	179.81	179.99
**AJ12**	161.76	166.35	160.22	179.89
**AJ13**	177.50	176.02	159.17	179.99
**AJ14**	176.48	176.11	179.37	179.79
**AJ15**	160.85	160.04	157.17	179.15
**AJ16**	175.98	175.93	150.62	171.24
**AJ17**	175.74	175.13	178.87	179.34
**AJ18**	157.79	158.89	157.77	179.88
**AJ19**	178.44	174.55	156.78	179.96
**AJ20**	176.41	174.49	179.68	179.96
**MS5**	145.18		144.06	

Table S2: Excited states properties (maximum wavelength in nm, oscillator strength, transition state and major transition of the FMOs with their contribution) of the isolated designed AJ dyes computed at the TD-OT-ωB97XD/6-31G(d,p) level of theory in THF solvent. The transition considered with $OS \geq 0.3$ and $\lambda_{max} \geq 370$ and contribution more than 15%.

Name	Transition	Major contribution (%)	λ_{max} (nm)	OS
**AJ1-BA**	$S_0 \rightarrow S_1$	$H \rightarrow L$ (51.2%), $H^{-1} \rightarrow L$ (44.3%)	433.97	0.8586
**AJ2-BA**	$S_0 \rightarrow S_1$	$H \rightarrow L$ (51.6%), $H^{-1} \rightarrow L$ (44.7%)	444.61	0.7227
**AJ3-BA**	$S_0 \rightarrow S_1$	$H \rightarrow L$ (84.5%), $H^{-1} \rightarrow L$ (14.2%)	794.52	0.4529
**AJ4-BA**	$S_0 \rightarrow S_1$	$H \rightarrow L$ (84.2%), $H^{-2} \rightarrow L$ (10.6%)	581.76	0.3704
**AJ5-BA**	$S_0 \rightarrow S_1$	$H \rightarrow L$ (84.2%), $H^{-1} \rightarrow L$ (12.0%)	736.42	0.4597
**AJ6-BA**	$S_0 \rightarrow S_1$	$H \rightarrow L$ (81.4%), $H^{-1} \rightarrow L$ (18.2%)	839.03	0.3672
**AJ7-BA**	$S_0 \rightarrow S_1$	$H \rightarrow L$ (80.2%), $H^{-2} \rightarrow L$ (11.4%)	513.07	0.5839
	$S_0 \rightarrow S_2$	$H^{-3} \rightarrow L$ (44.1%), $H^{-2} \rightarrow L$ (33.3%)	392.21	0.1571
**AJ8-BA**	$S_0 \rightarrow S_1$	$H \rightarrow L$ (77.9%)	538.38	0.5027
**AJ15-BA**	$S_0 \rightarrow S_1$	$H \rightarrow L$ (56.0%), $H^{-1} \rightarrow L$ (37.7%)	516.95	0.9488
**AJ16-BA**	$S_0 \rightarrow S_1$	$H \rightarrow L$ (63.7%), $H^{-2} \rightarrow L$ (25.0%)	501.84	0.9419
	$S_0 \rightarrow S_3$	$H \rightarrow L^{+1}$ (62.9%)	376.53	1.0004
**AJ17-BA**	$S_0 \rightarrow S_1$	$H \rightarrow L$ (63.9%), $H^{-2} \rightarrow L$ (25.6%)	517.38	1.0915
	$S_0 \rightarrow S_3$	$H \rightarrow L^{+2}$ (59.9%)	379.60	1.1715
**AJ18-BA**	$S_0 \rightarrow S_1$	$H^{-1} \rightarrow L$ (50.9%), $H \rightarrow L$ (41.9%)	521.82	1.4243
	$S_0 \rightarrow S_3$	$H \rightarrow L^{+2}$ (25.2%), $H^{-3} \rightarrow L$ (16.6%), $H^{-4} \rightarrow L$ (15.8%)	379.09	0.9678
**AJ19-BA**	$S_0 \rightarrow S_1$	$H^{-1} \rightarrow L$ (45.8%), $H \rightarrow L$ (44.1%)	514.97	1.6663
	$S_0 \rightarrow S_3$	$H \rightarrow L^{+2}$ (45.0%), $H \rightarrow L^{+1}$ (14.4%)	389.25	1.1882
**AJ20-BA**	$S_0 \rightarrow S_1$	$H \rightarrow L$ (47.5%), $H^{-1} \rightarrow L$ (41.1%)	516.36	1.6412
	$S_0 \rightarrow S_3$	$H \rightarrow L^{+2}$ (53.7%)	389.39	1.3318
**AJ1-CA**	$S_0 \rightarrow S_1$	$H^{-1} \rightarrow L$ (46.7%), $H \rightarrow L$ (33.2%)	474.22	0.9162
**AJ2-CA**	$S_0 \rightarrow S_1$	$H^{-1} \rightarrow L$ (47.0%), $H \rightarrow L$ (34.5%)	490.08	0.8404
**AJ3-CA**	$S_0 \rightarrow S_1$	$H \rightarrow L$ (83.7%), $H^{-1} \rightarrow L$ (14.0%)	805.09	0.5303
	$S_0 \rightarrow S_3$	$H \rightarrow L^{+1}$ (55.4%), $H^{-1} \rightarrow L^{+1}$ (32.1%)	419.18	0.5417
**AJ4-CA**	$S_0 \rightarrow S_1$	$H \rightarrow L$ (81.4%), $H^{-2} \rightarrow L$ (10.0%)	593.31	0.4761
	$S_0 \rightarrow S_4$	$H \rightarrow L^{+1}$ (57.4%), $H^{-2} \rightarrow L^{+1}$ (23.0%)	385.96	0.3021
**AJ5-CA**	$S_0 \rightarrow S_1$	$H \rightarrow L$ (85.3%)	747.61	0.5850
	$S_0 \rightarrow S_3$	$H \rightarrow L^{+1}$ (57.6%), $H^{-1} \rightarrow L^{+1}$ (25.4%)	406.85	0.5531
**AJ6-CA**	$S_0 \rightarrow S_1$	$H \rightarrow L$ (84.6%), $H^{-1} \rightarrow L$ (14.5%)	882.76	0.5083
	$S_0 \rightarrow S_3$	$H \rightarrow L^{+1}$ (60.5%), $H^{-1} \rightarrow L^{+1}$ (30.8%)	435.83	0.5581
**AJ7-CA**	$S_0 \rightarrow S_1$	$H \rightarrow L$ (72.0%)	524.36	0.7265
	$S_0 \rightarrow S_2$	$H^{-3} \rightarrow L$ (43.0%), $H^{-2} \rightarrow L$ (32.0%)	401.02	0.3869
**AJ8-CA**	$S_0 \rightarrow S_1$	$H \rightarrow L$ (72.7%)	549.67	0.6240
	$S_0 \rightarrow S_4$	$H \rightarrow L^{+2}$ (31.4%), $H \rightarrow L^{+1}$ (23.7%)	370.88	0.4328
**AJ15-CA**	$S_0 \rightarrow S_1$	$H \rightarrow L$ (61.6%), $H^{-2} \rightarrow L$ (18.2%)	550.50	1.0425

	$S_0 \rightarrow S_3$	$H \rightarrow L^{+1}$ (42.3%), $H^{-2} \rightarrow L^{+1}$ (21.5%), $H^{-3} \rightarrow L^{+1}$ (15.1%)	395.06	0.3774
**AJ16-CA**	$S_0 \rightarrow S_1$	$H \rightarrow L$ (65.9%), $H^{-2} \rightarrow L$ (17.1%)	516.75	0.8917
	$S_0 \rightarrow S_3$	$H \rightarrow L^{+2}$ (63.2%)	379.71	1.0388
**AJ17-CA**	$S_0 \rightarrow S_1$	$H \rightarrow L$ (60.5%)	552.29	1.0655
	$S_0 \rightarrow S_4$	$H \rightarrow L^{+2}$ (68.0%)	381.18	0.8891
**AJ18-CA**	$S_0 \rightarrow S_1$	$H \rightarrow L$ (43.4%), $H^{-1} \rightarrow L$ (24.5%)	537.45	1.5045
	$S_0 \rightarrow S_3$	$H^{-1} \rightarrow L^{+1}$ (20.4%)	396.99	0.6761
	$S_0 \rightarrow S_5$	$H \rightarrow L^{+2}$ (22.2%), $H^{-4} \rightarrow L$ (20.2%)	371.22	0.4499
	$S_0 \rightarrow S_6$	$H \rightarrow L^{+2}$ (27.9%)	369.82	0.3198
**AJ19-CA**	$S_0 \rightarrow S_1$	$H \rightarrow L$ (45.8%), $H^{-2} \rightarrow L$ (23.0%)	531.51	1.7528
	$S_0 \rightarrow S_3$	$H \rightarrow L^{+2}$ (37.5%)	396.41	1.2711
**AJ20-CA**	$S_0 \rightarrow S_1$	$H \rightarrow L$ (39.6%), $H^{-2} \rightarrow L$ (18.5%)	539.11	1.7431
	$S_0 \rightarrow S_4$	$H \rightarrow L^{+2}$ (57.3%), $H \rightarrow L$ (14.9%	392.80	1.0374

Table S3: Excited states properties (maximum wavelength in nm, oscillator strength, transition state and major transition of the FMOs with their contribution) of the isolated designed AJ dyes/TiO_2 computed at the TD-OT-ωB97XD/6-31G(d,p) level of theory in THF solvent. The transition considered with $OS \geq 0.03$ and $\lambda_{max} \geq 400$ and contribution more than 15%.

AJ@TiO_2	Transition	Major Contribution (%)	λ_{max} (nm)	OS
**AJ1-BA**	$S_0 \rightarrow S_1$	$H \rightarrow L$ (41.1%), $H^{-1} \rightarrow L$ (39.3%)	494.77	0.0423
	$S_0 \rightarrow S_3$	$H^{-1} \rightarrow L^{+2}$ (25.8%), $H \rightarrow L^{+2}$ (25.5%)	465.20	0.3121
	$S_0 \rightarrow S_4$	$H^{-1} \rightarrow L^{+3}$ (32.2%), $H \rightarrow L^{+3}$ (28.5%)	454.09	0.1959
	$S_0 \rightarrow S_6$	$H \rightarrow L^{+3}$ (16.6%), $H^{-1} \rightarrow L^{+3}$ (15.0%)	438.29	0.2504
	$S_0 \rightarrow S_9$	$H \rightarrow L^{+6}$ (30.1%), $H^{-1} \rightarrow L^{+6}$ (28.5%)	414.18	0.0740
**AJ2-BA**	$S_0 \rightarrow S_2$	$H^{-1} \rightarrow L^{+1}$ (41.0%), $H \rightarrow L^{+1}$ (38.8%)	511.55	0.0697
	$S_0 \rightarrow S_4$	$H^{-1} \rightarrow L^{+3}$ (36.2%), $H \rightarrow L^{+3}$ (33.7%)	481.17	0.2314
**AJ3-BA**	$S_0 \rightarrow S_1$	$H \rightarrow L$ (87.6%), $H^{-1} \rightarrow L$ (10.6%)	887.76	0.5617
	$S_0 \rightarrow S_4$	$H \rightarrow L^{+2}$ (74.5%), $H^{-1} \rightarrow L^{+2}$ (12.3%)	477.51	0.0563
	$S_0 \rightarrow S_{10}$	$H \rightarrow L^{+7}$ (58.5%)	412.50	0.0376
**AJ4-BA**	$S_0 \rightarrow S_1$	$H \rightarrow L^{+5}$ (76.6%)	633.02	0.4467
	$S_0 \rightarrow S_7$	$H^{-3} \rightarrow L^{+5}$ (38.5%), $H^{-2} \rightarrow L^{+5}$ (36.4%)	440.39	0.1070
**AJ5-BA**	$S_0 \rightarrow S_1$	$H \rightarrow L$ (72.2%)	787.45	0.5883
**AJ6-BA**	$S_0 \rightarrow S_1$	$H \rightarrow L$ (88.0%)	1057.07	0.4623
	$S_0 \rightarrow S_2$	$H^{-1} \rightarrow L$ (57.3%), $H^{-3} \rightarrow L$ (21.0%)	630.26	0.0931
**AJ7-BA**	$S_0 \rightarrow S_1$	$H \rightarrow L^{+4}$ (30.8%), $H \rightarrow L^{+5}$ (20.1%)	550.53	0.6373
**AJ8-BA**	$S_0 \rightarrow S_1$	$H \rightarrow L$ (83.7%)	689.49	0.4617
	$S_0 \rightarrow S_2$	$H^{-4} \rightarrow L$ (30.8%), $H^{-2} \rightarrow L$ (17.0%)	475.40	0.0418
	$S_0 \rightarrow S_3$	$H \rightarrow L^{+7}$ (19.9%), $H \rightarrow L^{+10}$ (19.0%)	458.13	0.1308
**AJ9-BA**	$S_0 \rightarrow S_1$	$H \rightarrow L$ (75.6%), $H^{-1} \rightarrow L$ (23.6%)	942.56	0.8763
	$S_0 \rightarrow S_2$	$H^{-1} \rightarrow L$ (65.3%), $H \rightarrow L$ (21.6%)	605.04	0.0394
	$S_0 \rightarrow S_4$	$H \rightarrow L^{+2}$ (61.8%), $H^{-1} \rightarrow L^{+2}$ (23.0%)	490.83	0.1284
	$S_0 \rightarrow S_6$	$H \rightarrow L^{+4}$ (64.6%), $H^{-1} \rightarrow L^{+4}$ (22.4%)	462.08	0.0664
	$S_0 \rightarrow S_8$	$H \rightarrow L^{+5}$ (42.8%)	441.90	0.1170
	$S_0 \rightarrow S_{10}$	$H \rightarrow L^{+7}$ (18.1%), $H \rightarrow L^{+8}$ (15.2%)	426.46	0.3866
**AJ10-BA**	$S_0 \rightarrow S_1$	$H \rightarrow L$ (74.5%), $H^{-1} \rightarrow L$ (25.2%)	882.01	0.7073
	$S_0 \rightarrow S_2$	$H^{-1} \rightarrow L$ (63.5%), $H \rightarrow L$ (22.5%)	575.36	0.0537
**AJ11-BA**	$S_0 \rightarrow S_1$	$H \rightarrow L$ (75.8%), $H^{-1} \rightarrow L$ (23.4%)	904.00	0.9288
	$S_0 \rightarrow S_2$	$H^{-1} \rightarrow L$ (64.4%), $H \rightarrow L$ (20.9%)	589.95	0.0328
	$S_0 \rightarrow S_4$	$H \rightarrow L^{+2}$ (56.8%), $H^{-1} \rightarrow L^{+2}$ (21.2%)	450.52	0.3001
	$S_0 \rightarrow S_7$	$H \rightarrow L^{+4}$ (49.3%), $H^{-1} \rightarrow L^{+4}$ (16.6%)	425.84	0.2596
	$S_0 \rightarrow S_8$	$H \rightarrow L^{+4}$ (17.6%), $H \rightarrow L^{+5}$ (15.1%)	410.69	0.5203
	$S_0 \rightarrow S_9$	$H \rightarrow L^{+5}$ (52.1%)	404.29	0.1029
	$S_0 \rightarrow S_{10}$	$H \rightarrow L^{+6}$ (52.4%)	400.49	0.1900
**AJ12-BA**	$S_0 \rightarrow S_1$	$H \rightarrow L$ (71.4%), $H^{-1} \rightarrow L$ (27.6%)	970.67	1.0605
	$S_0 \rightarrow S_2$	$H^{-1} \rightarrow L$ (59.1%), $H \rightarrow L$ (24.5%)	597.40	0.0560
	$S_0 \rightarrow S_3$	$H \rightarrow L^{+1}$ (62.2%), $H^{-1} \rightarrow L^{+1}$ (24.0%)	496.99	0.0619
	$S_0 \rightarrow S_7$	$H \rightarrow L^{+3}$ (50.5%), $H^{-1} \rightarrow L^{+3}$ (19.9%)	465.42	0.2692
	$S_0 \rightarrow S_9$	$H \rightarrow L^{+4}$ (52.5%), $H^{-1} \rightarrow L^{+4}$ (21.4%)	451.10	0.1707
**AJ13-BA**	$S_0 \rightarrow S_1$	$H \rightarrow L$ (82.3%), $H^{-1} \rightarrow L$ (16.5%)	954.53	1.0747
	$S_0 \rightarrow S_2$	$H^{-1} \rightarrow L$ (66.2%)	566.24	0.0809
	$S_0 \rightarrow S_4$	$H \rightarrow L^{+2}$ (61.1%), $H^{-1} \rightarrow L^{+2}$ (16.0%)	474.13	0.3408
	$S_0 \rightarrow S_8$	$H^{-5} \rightarrow L$ (30.6%), $H^{-3} \rightarrow L$ (25.5%)	450.25	0.1328
	$S_0 \rightarrow S_9$	$H \rightarrow L^{+4}$ (60.4%), $H^{-1} \rightarrow L^{+4}$ (14.5%)	445.94	0.1501

	$S_0 \rightarrow S_{10}$	$H \rightarrow L^{+5}$ (32.2%)	428.14	0.5806
**AJ14-BA**	$S_0 \rightarrow S_1$	$H \rightarrow L$ (73.5%), $H^{-1} \rightarrow L$ (25.2%)	957.19	1.1210
	$S_0 \rightarrow S_2$	$H^{-1} \rightarrow L$ (60.9%), $H \rightarrow L$ (22.2%)	588.52	0.0848
	$S_0 \rightarrow S_3$	$H \rightarrow L^{+1}$ (69.7%), $H^{-1} \rightarrow L^{+1}$ (21.8%)	496.49	0.0400
	$S_0 \rightarrow S_4$	$H \rightarrow L^{+2}$ (49.6%), $H^{-1} \rightarrow L^{+2}$ (18.2%)	469.85	0.5448
	$S_0 \rightarrow S_8$	$H \rightarrow L^{+4}$ (31.9%), $H \rightarrow L^{+2}$ (15.7%)	442.85	0.5200
	$S_0 \rightarrow S_{10}$	$H \rightarrow L^{+4}$ (34.7%)	428.08	0.5454
**AJ15-BA**	$S_0 \rightarrow S_1$	$H \rightarrow L^{+6}$ (37.9%), $H^{-1} \rightarrow L^{+6}$ (20.7%)	520.53	1.1131
	$S_0 \rightarrow S_2$	$H^{-1} \rightarrow L^{+6}$ (24.6%), $H \rightarrow L^{+6}$ (17.9%)	423.17	0.1928
**AJ16-BA**	$S_0 \rightarrow S_1$	$H \rightarrow L^{+1}$ (73.2%), $H^{-2} \rightarrow L^{+1}$ (17.3%)	551.60	0.8572
	$S_0 \rightarrow S_2$	$H^{-3} \rightarrow L^{+1}$ (44.4%), $H^{-2} \rightarrow L^{+1}$ (29.0%), $H \rightarrow L^{+1}$ (17.4%)	424.81	0.1239
**AJ17-BA**	$S_0 \rightarrow S_1$	$H \rightarrow L^{+4}$ (34.2%), $H \rightarrow L^{+3}$ (24.5%)	532.56	1.2143
	$S_0 \rightarrow S_3$	$H^{-3} \rightarrow L^{+4}$ (16.4%), $H^{-2} \rightarrow L^{+4}$ (15.9%)	419.74	0.2696
**AJ18-BA**	$S_0 \rightarrow S_1$	$H \rightarrow L^{+6}$ (33.2%), $H^{-1} \rightarrow L^{+6}$ (33.2%)	525.36	1.6624
	$S_0 \rightarrow S_3$	$H \rightarrow L^{+6}$ (23.2%), $H^{-1} \rightarrow L^{+6}$ (17.8%), $H^{-3} \rightarrow L^{+6}$ (17.7%)	421.39	0.0761
	$S_0 \rightarrow S_4$	$H \rightarrow L^{+1}$ (43.4%), $H^{-1} \rightarrow L^{+1}$ (21.7%)	401.33	0.1980
**AJ19-BA**	$S_0 \rightarrow S_1$	$H \rightarrow L^{+6}$ (29.1%), $H^{-1} \rightarrow L^{+6}$ (18.2%)	535.66	1.7981
	$S_0 \rightarrow S_3$	$H^{-3} \rightarrow L^{+6}$ (15.1%)	429.81	0.1004
	$S_0 \rightarrow S_4$	$H \rightarrow L^{+1}$ (58.6%)	406.97	0.1431
	$S_0 \rightarrow S_5$	$H^{-1} \rightarrow L$ (26.6%), $H^{-3} \rightarrow L$ (22.3%), $H \rightarrow L$ (20.4%)	400.27	0.0576
**AJ20-BA**	$S_0 \rightarrow S_1$	$H \rightarrow L^{+4}$ (33.2%), $H^{-1} \rightarrow L^{+4}$ (25.5%)	525.25	1.8019
	$S_0 \rightarrow S_2$	$H \rightarrow L^{+4}$ (18.4%), $H^{-1} \rightarrow L^{+4}$ (18.2%), $H^{-3} \rightarrow L^{+4}$ (16.2%)	418.63	0.0583
**AJ1-CA**	$S_0 \rightarrow S_1$	$H^{-1} \rightarrow L$ (30.8%), $H \rightarrow L$ (26.1%)	524.27	0.1695
	$S_0 \rightarrow S_2$	$H^{-1} \rightarrow L^{+3}$ (17.4%), $H^{-1} \rightarrow L^{+2}$ (12.4%)	515.10	0.4764
	$S_0 \rightarrow S_3$	$H \rightarrow L^{+1}$ (14.2%), $H^{-1} \rightarrow L^{+1}$ (13.4%)	485.93	0.0664
	$S_0 \rightarrow S_4$	$H \rightarrow L^{+1}$ (18.8%), $H \rightarrow L$ (16.4%), $H^{-1} \rightarrow L^{+1}$ (16.0%),	478.93	0.0576
**AJ2-CA**	$S_0 \rightarrow S_1$	$H \rightarrow L$ (34.4%), $H^{-1} \rightarrow L$ (33.1%)	536.68	0.0309
	$S_0 \rightarrow S_2$	$H^{-1} \rightarrow L^{+3}$ (11.5%)	517.31	0.5574
	$S_0 \rightarrow S_3$	$H^{-1} \rightarrow L^{+2}$ (21.8%), $H \rightarrow L^{+2}$ (18.3%)	502.65	0.0492
	$S_0 \rightarrow S_9$	$H \rightarrow L^{+2}$ (32.7%)	434.32	0.0308
	$S_0 \rightarrow S_{10}$	$H^{-1} \rightarrow L^{+2}$ (24.2%)	431.02	0.0522
**AJ3-CA**	$S_0 \rightarrow S_1$	$H \rightarrow L$ (86.9%), $H^{-1} \rightarrow L$ (11.2%)	897.4	0.6186
	$S_0 \rightarrow S_6$	$H \rightarrow L^{+4}$ (40.6%), $H^{-1} \rightarrow L^{+4}$ (10.6%)	469.89	0.2871
	$S_0 \rightarrow S_7$	$H \rightarrow L^{+5}$ (26.6%), $H \rightarrow L^{+7}$ (22.0%)	449.53	0.1378
	$S_0 \rightarrow S_8$	$H \rightarrow L^{+5}$ (23.3%), $H \rightarrow L^{+7}$ (13.9%), $H \rightarrow L^{+4}$ (10.4%)	441.08	0.0304
**AJ4-CA**	$S_0 \rightarrow S_1$	$H \rightarrow L^{+4}$ (44.2%), $H \rightarrow L^{+3}$ (22.1%),	639.03	0.5709
	$S_0 \rightarrow S_5$	$H \rightarrow L^{+3}$ (27.9%), $H \rightarrow L^{+4}$ (16.7%)	453.11	0.1684
	$S_0 \rightarrow S_6$	$H^{-2} \rightarrow L^{+4}$ (21.8%), $H^{-3} \rightarrow L^{+4}$ (17.1%), $H \rightarrow L^{+3}$ (15.8%)	449.27	0.1458
	$S_0 \rightarrow S_8$	$H \rightarrow L^{+7}$ (23.1%), $H \rightarrow L^{+4}$ (22.6%)	428.88	0.0391
**AJ5-CA**	$S_0 \rightarrow S_1$	$H \rightarrow L$ (88.7%), $H^{-1} \rightarrow L$ (8.2%)	869.58	0.6137

	$S_0 \rightarrow S_4$	$H \rightarrow L^{+2}$ (39.5%), $H^{-2} \rightarrow L^{+2}$ (10.0%)	448.28	0.1231
	$S_0 \rightarrow S_5$	$H \rightarrow L^{+1}$ (29.8%), $H \rightarrow L^{+3}$ (11.3%)	428.68	0.1156
	$S_0 \rightarrow S_7$	$H \rightarrow L^{+5}$ (24.2%)	412.92	0.0773
**AJ6-CA**	$S_0 \rightarrow S_1$	$H \rightarrow L$ (90.1%), $H^{-1} \rightarrow L$ (11.0%)	1062.51	0.5645
	$S_0 \rightarrow S_2$	$H^{-1} \rightarrow L$ (71.4%), $H^{-3} \rightarrow L$ (13.3%)	634.84	0.0803
	$S_0 \rightarrow S_3$	$H \rightarrow L^{+1}$ (37.4%), $H \rightarrow L^{+2}$ (26.2%)	535.73	0.0323
	$S_0 \rightarrow S_4$	$H \rightarrow L^{+4}$ (39.0%), $H \rightarrow L^{+3}$ (22.1%)	525.56	0.1376
	$S_0 \rightarrow S_6$	$H \rightarrow L^{+6}$ (20.5%), $H \rightarrow L^{+8}$ (16.9%)	479.50	0.2315
	$S_0 \rightarrow S_7$	$H \rightarrow L^{+5}$ (29.8%), $H \rightarrow L^{+8}$ (14.8%)	470.46	0.0646
	$S_0 \rightarrow S_9$	$H \rightarrow L^{+7}$ (27.8%), $H \rightarrow L^{+9}$ (16.3%)	456.24	0.0383
**AJ7-CA**	$S_0 \rightarrow S_1$	$H \rightarrow L^{+5}$ (33.5%), $H \rightarrow L^{+14}$ (11.4%)	555.53	0.7718
	$S_0 \rightarrow S_5$	$H \rightarrow L^{+3}$ (61.7%)	428.67	0.0433
	$S_0 \rightarrow S_6$	$H \rightarrow L^{+4}$ (45.3%), $H \rightarrow L^{+5}$ (13.3%)	413.94	0.1623
	$S_0 \rightarrow S_7$	$H \rightarrow L^{+4}$ (16.2%), $H^{-3} \rightarrow L^{+5}$ (14.9%)	411.14	0.4065
**AJ8-CA**	$S_0 \rightarrow S_1$	$H \rightarrow L$ (83.9%)	711.53	0.4677
	$S_0 \rightarrow S_2$	$H^{-4} \rightarrow L$ (34.0%), $H^{-2} \rightarrow L$ (14.2%)	478.96	0.0427
	$S_0 \rightarrow S_3$	$H \rightarrow L^{+8}$ (17.1%), $H^{-4} \rightarrow L$ (14.6%)	466.83	0.1653
	$S_0 \rightarrow S_4$	$H \rightarrow L^{+1}$ (42.6%)	413.63	0.1636
**AJ9-CA**	$S_0 \rightarrow S_1$	$H \rightarrow L$ (79.4%), $H^{-1} \rightarrow L$ (19.6%)	943.78	1.2206
	$S_0 \rightarrow S_2$	$H^{-1} \rightarrow L$ (66.7%), $H \rightarrow L$ (18.0%)	623.63	0.0592
	$S_0 \rightarrow S_4$	$H \rightarrow L^{+2}$ (33.9%), $H^{-1} \rightarrow L^{+2}$ (21.0%), $H \rightarrow L^{+3}$ (19.5%)	516.41	0.0472
	$S_0 \rightarrow S_6$	$H \rightarrow L^{+4}$ (41.7%), $H^{-1} \rightarrow L^{+4}$ (24.2%)	493.57	0.3569
	$S_0 \rightarrow S_7$	$H \rightarrow L^{+5}$ (29.7%), $H^{-1} \rightarrow L^{+5}$ (17.5%)	469.67	0.0306
	$S_0 \rightarrow S_8$	$H \rightarrow L^{+5}$ (16.9%), $H \rightarrow L^{+7}$ (14.3%)	464.5	0.2665
	$S_0 \rightarrow S_9$	$H \rightarrow L^{+6}$ (47.1%), $H^{-1} \rightarrow L^{+6}$ (27.3%)	456.38	0.0385
**AJ10-CA**	$S_0 \rightarrow S_1$	$H \rightarrow L$ (77.0%), $H^{-1} \rightarrow L$ (21.5%)	931.02	1.2381
	$S_0 \rightarrow S_2$	$H^{-1} \rightarrow L$ (61.9%), $H \rightarrow L$ (19.6%)	622.75	0.0310
	$S_0 \rightarrow S_3$	$H \rightarrow L^{+1}$ (43.4%), $H^{-1} \rightarrow L^{+1}$ (28.5%)	509.13	0.0484
	$S_0 \rightarrow S_5$	$H \rightarrow L^{+3}$ (21.3%), $H \rightarrow L^{+4}$ (17.9%)	482.54	0.3032
	$S_0 \rightarrow S_9$	$H \rightarrow L^{+5}$ (13.9%), $H \rightarrow L^{+3}$ (11.9%)	444.98	0.1963
**AJ11-CA**	$S_0 \rightarrow S_1$	$H \rightarrow L$ (76.6%), $H^{-1} \rightarrow L$ (20.5%)	920.99	1.1472
	$S_0 \rightarrow S_4$	$H \rightarrow L^{+2}$ (33.6%), $H^{-1} \rightarrow L^{+2}$ (22.6%)	472.54	0.4455
	$S_0 \rightarrow S_7$	$H \rightarrow L^{+5}$ (25.5%), $H^{-1} \rightarrow L^{+5}$ (14.8%)	425.32	0.1025
	$S_0 \rightarrow S_8$	$H \rightarrow L^{+4}$ (24.0%)	418.55	0.0510
**AJ12-CA**	$S_0 \rightarrow S_1$	$H \rightarrow L$ (67.2%), $H^{-1} \rightarrow L$ (31.0%)	965.38	1.3838
	$S_0 \rightarrow S_2$	$H^{-1} \rightarrow L$ (56.6%), $H \rightarrow L$ (28.7%)	628.4	0.0649
	$S_0 \rightarrow S_3$	$H \rightarrow L^{+1}$ (36.1%), $H^{-1} \rightarrow L^{+1}$ (30.1%)	521.38	0.1180
	$S_0 \rightarrow S_5$	$H \rightarrow L^{+3}$ (20.2%), $H^{-1} \rightarrow L^{+3}$ (17.7%)	498.41	0.4999
	$S_0 \rightarrow S_7$	$H^{-4} \rightarrow L$ (16.7%), $H^{-7} \rightarrow L$ (14.8%),	477.34	0.1448

	$S_0 \rightarrow S_8$	$H^{-4} \rightarrow L$ (15.4%), $H^{-7} \rightarrow L$ (13.6%)	474.54	0.2732
**AJ13-CA**	$S_0 \rightarrow S_1$	$H \rightarrow L$ (70.7%), $H^{-1} \rightarrow L$ (27.1%)	958.67	1.4544
	$S_0 \rightarrow S_2$	$H^{-1} \rightarrow L$ (57.9%), $H \rightarrow L$ (24.6%)	615.21	0.0943
	$S_0 \rightarrow S_3$	$H \rightarrow L^{+1}$ (18.4%)	506.97	0.7596
	$S_0 \rightarrow S_4$	$H \rightarrow L^{+1}$ (14.1%), $H \rightarrow L^{+3}$ (13.8%)	487.09	0.1175
	$S_0 \rightarrow S_8$	$H \rightarrow L^{+6}$ (24.9%), $H^{-1} \rightarrow L^{+6}$ (20.0%)	455.42	0.1990
	$S_0 \rightarrow S_9$	$H \rightarrow L^{+5}$ (37.1%), $H^{-1} \rightarrow L^{+5}$ (28.8%)	444.28	0.0352
**AJ14-CA**	$S_0 \rightarrow S_1$	$H \rightarrow L$ (73.3%), $H^{-1} \rightarrow L$ (24.4%)	952.77	1.4099
	$S_0 \rightarrow S_2$	$H^{-1} \rightarrow L$ (59.3%), $H \rightarrow L$ (22.1%)	601.28	0.1217
	$S_0 \rightarrow S_3$	$H \rightarrow L^{+4}$ (18.6%)	514.01	0.8802
	$S_0 \rightarrow S_4$	$H \rightarrow L^{+1}$ (40.2%), $H^{-1} \rightarrow L^{+1}$ (20.1%),	482.97	0.0727
	$S_0 \rightarrow S_8$	$H \rightarrow L^{+5}$ (28.9%), $H \rightarrow L^{+3}$ (17.7%), $H^{-1} \rightarrow L^{+5}$ (15.8%)	450.31	0.0355
**AJ15-CA**	$S_0 \rightarrow S_1$	$H \rightarrow L$ (72.0%), $H^{-2} \rightarrow L$ (12.2%),	629.97	0.9364
	$S_0 \rightarrow S_2$	$H^{-3} \rightarrow L$ (46.4%), $H^{-2} \rightarrow L$ (22.1%), $H \rightarrow L$ (17.9%)	473.76	0.3998
	$S_0 \rightarrow S_3$	$H \rightarrow L^{+7}$ (15.7%), $H \rightarrow L^{+5}$ (14.5%)	426.15	0.3856
	$S_0 \rightarrow S_4$	$H \rightarrow L^{+1}$ (72.3%)	406.36	0.0681
**AJ16-CA**	$S_0 \rightarrow S_1$	$H \rightarrow L^{+1}$ (35.3%), $H \rightarrow L$ (18.9%)	574.29	1.1714
	$S_0 \rightarrow S_3$	$H^{-3} \rightarrow L^{+1}$ (25.4%), $H^{-3} \rightarrow L$ (16.6%)	439.30	0.5006
**AJ17-CA**	$S_0 \rightarrow S_1$	$H \rightarrow L$ (72.6%), $H^{-2} \rightarrow L$ (9.8%)	635.33	0.9805
	$S_0 \rightarrow S_2$	$H^{-3} \rightarrow L$ (43.2%), $H^{-2} \rightarrow L$ (18.5%), $H \rightarrow L$ (16.3%)	466.12	0.4433
	$S_0 \rightarrow S_3$	$H \rightarrow L^{+4}$ (26.6%), $H \rightarrow L^{+3}$ (14.0%)	428.86	0.2532
	$S_0 \rightarrow S_4$	$H \rightarrow L^{+1}$ (63.7%), $H \rightarrow L^{+23}$ (12.3%)	400.88	0.2266
**AJ18-CA**	$S_0 \rightarrow S_1$	$H \rightarrow L^{+1}$ (33.3%), $H^{-1} \rightarrow L^{+1}$ (17.6%)	561.42	1.6926
	$S_0 \rightarrow S_2$	$H \rightarrow L$ (42.1%), $H^{-1} \rightarrow L$ (13.9%)	455.32	0.0470
	$S_0 \rightarrow S_3$	$H \rightarrow L^{+1}$ (18.9%), $H^{-3} \rightarrow L^{+1}$ (17.4%)	451.23	0.3779
	$S_0 \rightarrow S_4$	$H \rightarrow L^{+1}$ (23.9%), $H \rightarrow L^{+3}$ (17.7%)	427.19	0.0362
	$S_0 \rightarrow S_7$	$H \rightarrow L^{+3}$ (27.4%)	417.41	0.1098
**AJ19-CA**	$S_0 \rightarrow S_1$	$H \rightarrow L^{+1}$ (39.4%), $H^{-2} \rightarrow L^{+1}$ (16.2%)	571.12	1.9198
	$S_0 \rightarrow S_3$	$H^{-3} \rightarrow L^{+1}$ (29.2%), $H \rightarrow L^{+1}$ (15.6%), $H^{-2} \rightarrow L^{+1}$ (15.5%)	451.21	0.3890
	$S_0 \rightarrow S_7$	$H \rightarrow L^{+3}$ (17.6%), $H \rightarrow L^{+4}$ (13.0%),	417.78	0.1116
**AJ20-CA**	$S_0 \rightarrow S_1$	$H \rightarrow L$ (44.1%), $H \rightarrow L^{+5}$ (11.6%)	572.33	1.7442
	$S_0 \rightarrow S_2$	$H \rightarrow L$ (29.7%), $H^{-3} \rightarrow L$ (23.2%)	448.05	0.3857

Table S4: Adsorption energy (E_{ads}) in eV, change in LUMO energy before and after the adsorption in eV, λ_{max} in nm of the designed dyes/TiO_2 complexes, and exciton binding energy (E_b) in eV. All the calculations are performed in THF solvent.

Name	E_{ads}		$^{a}\Delta E_{LUMO}$		λ_{max}		ΔG_{inj} (eV)		E_b	
	BA	CA	BA	CA	BA	CA	BA	CA	BA	CA
AJ1	-0.63	-0.88	0.94	0.69	494.78	524.27	-1.78	-1.51	0.2	-0.22
AJ2	-1.96	-2.02	0.95	0.83	552.28	536.67	-1.71	-1.44	0.19	-0.16
AJ3	-1.99	-2.14	0.04	0.07	887.76	897.37	-0.44	-0.35	0.06	0.04
AJ4	-0.99	-0.87	0.13	0.43	633.04	639.04	-1.02	-0.95	0.06	-0.01
AJ5	-0.98	-0.68	0.04	0.11	787.46	869.60	-0.52	-0.42	0.11	0.13
AJ6	-1.68	-1.99	0.03	-0.09	1057.09	1062.53	-0.37	-0.27	-0.05	-0.02
AJ7	-0.35	-0.87	0.28	0.62	550.51	555.53	-1.26	-1.19	-0.08	-0.16
AJ8	-1.66	-1.94	0.07	0.24	689.48	711.54	-1.16	-1.09	-0.18	-0.23
AJ15	-0.37	-0.65	0.07	0.41	520.54	629.98	-1.24	-1.13	-0.23	-0.39
AJ16	-0.38	-0.73	0.17	0.38	551.61	574.29	-1.22	-1.20	-0.23	-0.28
AJ17	-0.62	-0.57	0.06	0.34	532.56	635.34	-1.13	-1.02	-0.26	-0.47
AJ18	-0.41	-0.35	0.05	0.28	525.35	561.43	-1.23	-1.2	-0.26	-0.46
AJ19	-0.66	-0.55	0.10	0.25	535.65	571.12	-1.17	-1.13	-0.19	-0.42
AJ20	-0.38	-0.33	0.04	0.23	525.25	572.33	-1.15	-1.09	-0.26	-0.50
Ref	-1.99		0.59		473.89		-1.51		0.02	

$^{a}\Delta E_{LUMO} = E_{LUMO}^{iolated\ dye} - E_{LUMO}^{dye@TiO_2}$

References:

(1) Vetere, V.; Adamo, C.; Maldivi, P. Performance of the `parameter Free' PBE0 Functional for the Modeling of Molecular Properties of Heavy Metals. *Chem Phys Lett* **2000**, *325* (1–3), 99–105. https://doi.org/10.1016/S0009-2614(00)00657-6.

(2) Payne, A.-J.; Rice, N. A.; McAfee, S. M.; Li, S.; Josse, P.; Cabanetos, C.; Risko, C.; Lessard, B. H.; Welch, G. C. Donor or Acceptor? How Selection of the Rylene Imide End Cap Impacts the Polarity of π-Conjugated Molecules for Organic Electronics. *ACS Appl Energy Mater* **2018**, *1* (9), 4906–4916. https://doi.org/10.1021/acsaem.8b00929.

(3) Vijayan, S.; Sparks, N.; Roy, J.; Smith, C.; Tate, C.; Hammer, N.; Leszczynski, J.; Watkins, D. Evaluating Donor Effects in Isoindigo-Based Small Molecular Fluorophores. *J Phys Chem A* **2020**, *124* (51), 10777–10786. https://doi.org/10.1021/acs.jpca.0c07796.

(4) Souza, J. P. A.; Benatto, L.; Candiotto, G.; Wouk, L.; Koehler, M. Dynamics of Vibrationally Coupled Intersystem Crossing in State-of-the-Art Organic Optoelectronic Materials. *Commun Chem* **2025**, *8* (1), 1–15. https://doi.org/10.1038/S42004-025-01485-3.

(5) Shen, Z.; Eickemeyer, F. T.; Gao, J.; Pfeifer, L.; Bradford, D.; Freitag, M.; Zakeeruddin, S. M.; Grätzel, M. Molecular Engineering of Low-Cost, Efficient, and Stable Photosensitizers for Dye-Sensitized Solar Cells. *Chem* **2023**, *9* (12), 3637–3647. https://doi.org/10.1016/j.chempr.2023.08.013.

(6) Zhang, D.; Stojanovic, M.; Ren, Y.; Cao, Y.; Eickemeyer, F. T.; Socie, E.; Vlachopoulos, N.; Moser, J. E.; Zakeeruddin, S. M.; Hagfeldt, A.; Grätzel, M. A Molecular Photosensitizer Achieves a Voc of 1.24 V Enabling Highly Efficient and Stable Dye-Sensitized Solar Cells with Copper(II/I)-Based Electrolyte. *Nature Communications 2021 12:1* **2021**, *12* (1), 1–10. https://doi.org/10.1038/s41467-021-21945-3.

(7) Ren, Y.; Zhang, D.; Suo, J.; Cao, Y.; Eickemeyer, F. T.; Vlachopoulos, N.; Zakeeruddin, S. M.; Hagfeldt, A.; Grätzel, M. Hydroxamic Acid Pre-Adsorption Raises the Efficiency of Cosensitized Solar Cells. *Nature* **2023**, *613* (7942), 60–65. https://doi.org/10.1038/s41586-022-05460-z.

(8) Hutchison, G. R.; Ratner, M. A.; Marks, T. J. Hopping Transport in Conductive Heterocyclic Oligomers: Reorganization Energies and Substituent Effects. *J Am Chem Soc* **2005**, *127* (7), 2339–2350. https://doi.org/10.1021/ja0461421.
